# The aryl hydrocarbon receptor controls mesenchymal stromal cell-mediated immunomodulation via ubiquitination of eukaryotic elongation factor-2 kinase

**DOI:** 10.1038/s41419-023-06341-7

**Published:** 2023-12-09

**Authors:** Enkhmaa Lkhagva-Yondon, Myeong Seong Seo, Yena Oh, Jonghun Jung, Eunhae Jeon, Kwangmin Na, Hyun Seung Yoo, Woo Chul Kim, Charlotte Esser, Sun U. Song, Myung-Shin Jeon

**Affiliations:** 1 Translational Research Center, Inha University Hospital, Incheon, 22332 Republic of Korea; 2 Program in Biomedical Science & Engineering Inha University, Incheon, 22212 Republic of Korea; 3 Department of Molecular Biomedicine, College of Medicine Inha University, Incheon, 22212 Republic of Korea; 4 Department of Radiation Oncology, Inha University Hospital, Incheon, 22332 Republic of Korea; 5 IUF-Leibniz Research Institute for Environmental Medicine, Düsseldorf, 40021 Germany; 6 SCM Lifescience, Incheon, 21999 Republic of Korea

**Keywords:** Mesenchymal stem cells, Stem-cell research

## Abstract

Mesenchymal stem cells (MSCs) have great therapeutic advantages due to their immunosuppressive properties. The aryl hydrocarbon receptor (AHR) is a ligand-activated transcription factor whose signaling plays an important role in the immune system. AHR may be involved in the regulation of MSC-associated immunomodulatory functions. However, the mechanisms by which AHR controls the immunosuppressive functions of MSCs are not well understood. Here, we report that *Ahr*-deficient MSCs show decreased therapeutic efficacy against graft-versus-host disease (GVHD) compared to wild-type (WT)-MSCs. This was probably due to decreased iNOS protein expression, which is a key regulatory enzyme in MSC immunomodulation. The expression of eukaryotic elongation factor 2 kinase (eEF2K), which inhibits the elongation stage of protein synthesis, is significantly increased in the *Ahr*-deficient MSCs. Inhibition of eEF2K restored iNOS protein expression. AHR is known to act as an E3 ligase together with CUL4B. We observed constitutive binding of AHR to eEF2K. Consequently, ubiquitination and degradation of eEF2K were inhibited in *Ahr*-deficient MSCs and by the AHR antagonist CH223191 in WT-MSCs. In summary, AHR regulates the immunomodulatory functions of MSCs through ubiquitination of eEF2K, thereby controlling iNOS protein synthesis and its product, nitric oxide levels.

## Introduction

Mesenchymal stem cells (MSCs) are stromal cells that can self-renew and differentiate into osteocytes, adipocytes, chondrocytes, hepatocytes, myocytes, and neurons [1–4]. MSCs have been investigated in cell therapies for immune-mediated inflammatory diseases such as graft-versus-host disease (GVHD) because of their anti-inflammatory and immunosuppressive properties [5–8]. The immunosuppressive function of murine MSCs is elicited by the pro-inflammatory cytokine IFN-γ with TNF-α and IL-1β [5]. These cytokines induce inducible nitric oxide synthase (iNOS) in murine MSCs, which suppresses T-cell responsiveness through nitric oxide (NO) [5, 9, 10].

The aryl hydrocarbon receptor (AHR) is a ligand-induced transcription factor that regulates multiple functions such as inflammatory processes, adhesion, proliferation, differentiation, endocrine disruption, and degradation of xenobiotic molecules [11]. A variety of ligands activate AHR, including xenobiotics, tryptophan (Trp) metabolites, microbiome metabolites, and endogenous molecules [12]. In the absence of a ligand, AHR remains in a cytoplasmic multiprotein complex that includes HSP90, XAP2, c-Src, and the co-chaperone p-23 (p23). Upon ligand binding, the complex dissolves and AHR translocates into the nucleus, dimerizing with the AHR nuclear translocator (ARNT). The AHR-ARNT complex then initiates transcription of target genes harboring xenobiotic-responsive elements (XRE) [12]. A well-known target gene is cytochrome P450 *Cyp1a1* [11, 13]. In addition, AHR can also act as an E3 ubiquitin ligase in the cytosol, which targets substrates for ubiquitination and degradation by the proteasome, e.g., estrogen receptor alpha (ERɑ), cellular retinoic acid binding proteins (CRABPs), and peroxisome proliferator-activated receptor (PPARγ) [14–17].

MSCs express AHR and show increased expression of the target genes *Cyp1a1* and *Cyp1b1* upon treatment with AHR-specific agonists (TCDD, FICZ, derivatives of Trp, and benzo(a)pyrene) [18, 19]. Several studies have reported that ligand-activated AHR plays a role in the anti-inflammatory and immunomodulatory effects of MSCs [20–23]. A metabolite of Trp, kynurenine-pretreated MSCs together with IFN-γ and TGF-β upregulate immunomodulatory genes in MSCs, such as *Nos2*, *Ido*, *Ptgs2*, *Hmox1*, *Pge2*, *Lif*, and *CD274*. The administration of those cytokine-pretreated MSCs decreased GVHD scores and improved mouse survival compared with untreated MSCs [20]. However, the exact molecular mechanisms underlying the involvement of AHR in MSC-induced immunosuppression have not been thoroughly investigated.

Here, using MSCs from *Ahr-*deficient mice, we demonstrated decreased iNOS protein expression and NO production which reduced the inhibition of T-cell proliferation and decreased the therapeutic efficacy in GVHD compared to wild-type (WT)-MSCs. As a molecular mechanism, we found for the first time that the decreased iNOS protein expression in *Ahr-*deficient MSCs (KO-MSCs) was due to the increased eukaryotic elongation factor 2 kinase (eEF2K), which is a negative regulator of mRNA translation elongation. Ubiquitination and degradation of eEF2K are decreased in KO-MSCs. As an E3 ligase, AHR is probably involved in the immunomodulatory functions of MSCs via regulating eEF2K ubiquitination.

## Results

### Efficacy of *Ahr* KO-MSCs on GVHD treatment compared to WT-MSCs

**Fig. 1:**
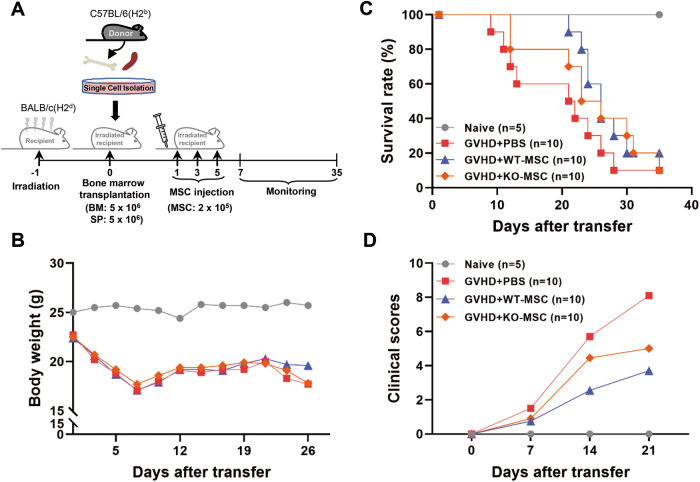
Effect of *Ahr*^*−/*^^−^ MSC on GVHD treatment. **A** To induce GVHD, BALB/c mice were irradiated with a dose of 8.5 Gy. One day after irradiation, 5 × 10^6^ bone marrow and 5 × 10^6^ spleen cells from C57BL/6 mice were injected. On days 1, 3, and 5 after transplantation, PBS, 2 × 10^5^ WT-, and KO-MSCs were injected intravenously to observe the therapeutic efficacy until 35 days. Bone marrow was injected as a negative control, naïve group (*n* = 5). PBS (*n* = 10), WT-MSCs (*n* = 10), and KO-MSCs (*n* = 10) were injected into the induced GVHD mice. The weight changes (**B**) and survival rate (**C**) of the GVHD mice were examined over the experimental period. **D** The clinical scores were monitored according to the severity of symptoms such as ruffled hair, hunched back, diarrhea, damaged skin, and ascites.

As MSCs possess therapeutic efficacy against GVHD [5, 7], we evaluated whether *Ahr* KO-MSCs could affect the therapeutic efficacy of GVHD in vivo. MSCs were injected on days 1, 3, and 5 after GVHD induction (Fig. 1A). We examined the survival rate for up to 35 days after MSC administration. The body weights of mice were similar (Fig. 1B). However, mice that received WT-MSCs had a prolonged survival rate compared to the PBS group, while KO-MSCs showed a reduced survival rate compared to WT-MSCs. Still, better than the PBS group (Fig. 1C). Clinical scores showed similar results to survival (Fig. 1D). These data suggest that AHR in MSCs might be involved in the therapeutic efficacy against GVHD.

### Effect of *Ahr* KO-MSCs on suppression of T-cell activation

**Fig. 2:**
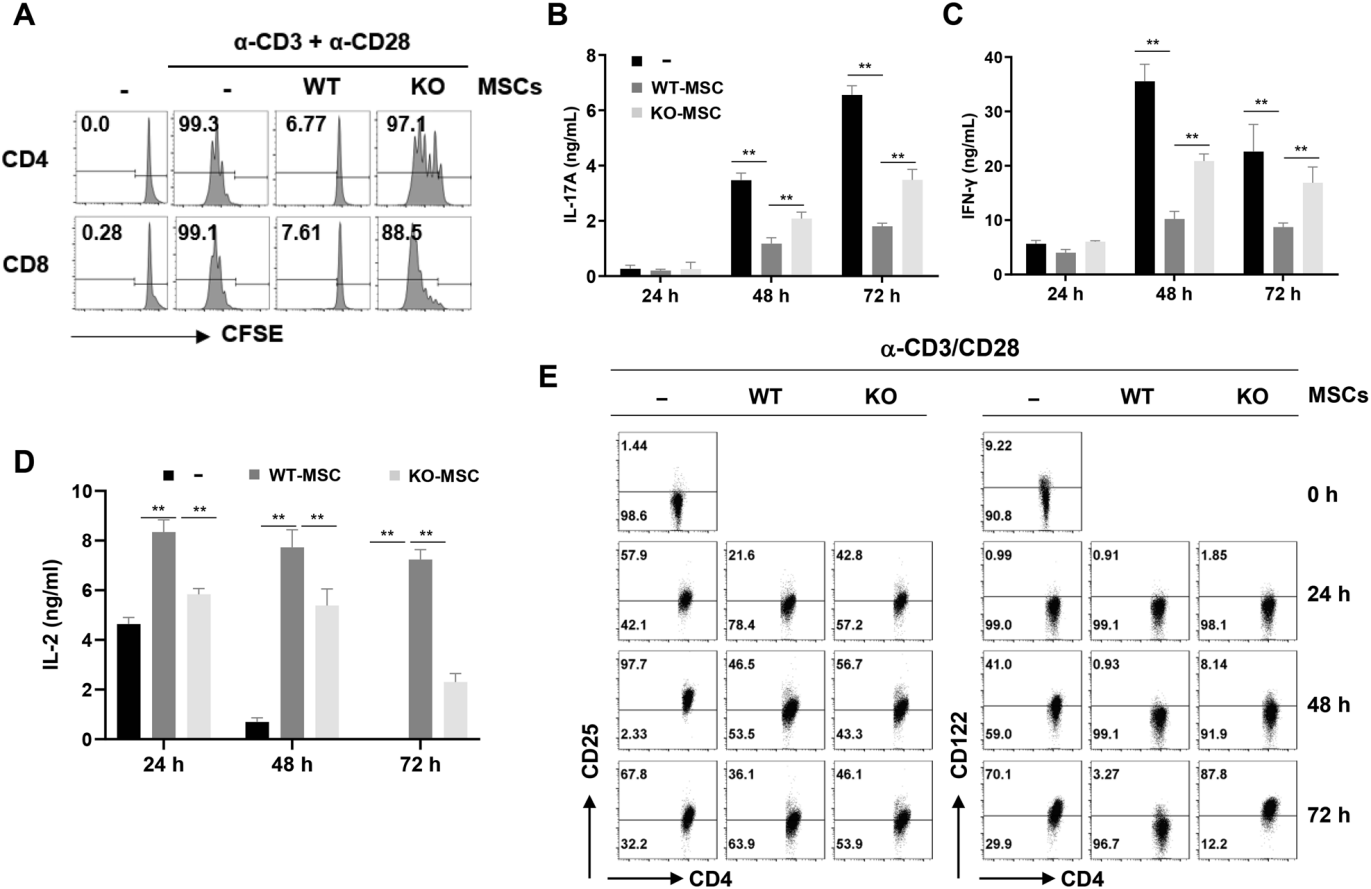
Effect of *Ahr*^*−/*^^−^ MSC on T cell activation. **A** Isolated splenocytes from WT mice were stimulated with anti-CD3 and anti-CD28 antibodies in the presence of WT- and KO-MSCs. After 3 days, CD4 and CD8 T cell divisions were analyzed by CFSE. **B** IL-17A, (**C**) IFN-γ, and (**D**) IL-2 levels in the culture media were measured by ELISA. **E** Expression of CD25 and CD122 on CD4 T cells was analyzed by flow cytometry. All experiments were repeated at least twice, and similar results were obtained. The statistical *p*-value between the comparison of multiple groups was evaluated by a two-way ANOVA followed by Tukey’s multiple comparison test. **P-values = ≤ 0.001 were considered statistically significant.

The therapeutic efficacy of MSCs against GVHD is mediated by the immunosuppressive functions of MSCs, particularly the inhibition of T-cell activation [5]. Therefore, we investigated whether *Ahr* deficiency affects T-cell activation. To measure T-cell proliferation, lymphocytes were stained with CFSE and stimulated with anti-CD3 and anti-CD28 antibodies in the presence or absence of MSCs. T-cell divisions were significantly inhibited by WT-MSCs, whereas KO-MSCs did not effectively inhibit T-cell proliferation (Fig. 2A). In addition, KO-MSCs showed less inhibition of IFN-γ and IL-17A production than did WT-MSCs (Fig. 2B, C). Previously, we reported increased IL-2 detection in the co-cultured media of T cells with MSCs because of the inhibition of IL-2 receptor (CD25) expression on T cells via the mTOR pathway. This suppression of CD25 expression is mediated by secreted NO in MSCs and is an indicator of the immunosuppressive function of MSCs [24]. Here, we compared the expression of IL-2 receptors and IL-2 expression in lymphocyte co-cultures of either WT or KO-MSCs. In the presence of KO-MSCs, fewer decreased IL-2 receptors and less increased IL-2 detection were observed on the co-cultured T cells compared to the WT-MSCs (Fig. 2D, E). These results suggest that the deletion of the *Ahr* gene in MSCs reduced the immunosuppressive function of MSCs.

### *Ahr* KO- MSCs produced less NO via decreased iNOS protein expression

**Fig. 3:**
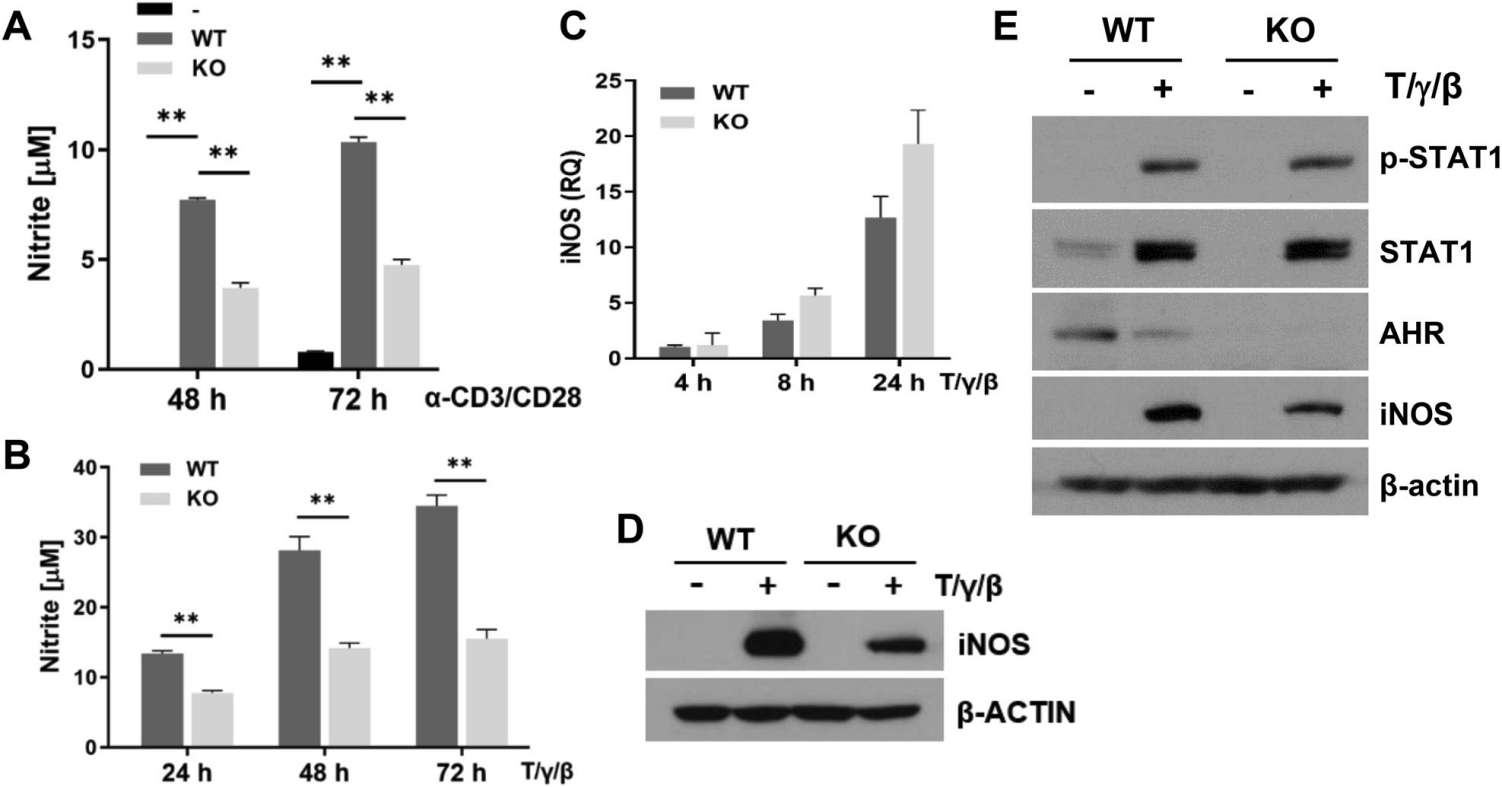
*Ahr* KO-MSCs produced less NO due to decreased iNOS protein expression. **A** NO production was measured in anti-CD3 and anti-CD28 stimulated splenocyte culture media. The statistical *p*-value for the comparison of multiple groups was evaluated by a two-way ANOVA with Bonferroni’s multiple comparison test. **B** WT- and KO-MSCs were stimulated by TNF-α, IFN-γ, and IL-1β. NO production was measured in the culture media. **C** iNOS mRNA levels were measured by qRT-PCR. (**D**) iNOS, (**E**) STAT1, and p-STAT1 protein levels were measured by western blotting. All experiments were performed at least twice, and representative results were presented. The statistical *p*-value between the comparison of multiple groups was evaluated by a two-way ANOVA followed by Tukey’s multiple comparison test. **P-values = ≤0.001 were considered statistically significant.

NO is a significant soluble factor for MSC-mediated immunosuppression [5, 9, 24]. Therefore, we measured the production of NO in the co-culture media of lymphocytes and MSCs. WT-MSCs increased NO production compared to lymphocytes alone, while KO-MSCs increased to a lesser extent (Fig. 3A). Since the production of NO is strongly induced in MSCs by pro-inflammatory cytokine stimulation [5], we stimulated MSCs with TNF-α, IFN-γ, and IL-1β. Consistent with the results of co-culture with lymphocytes, KO-MSCs produced less NO than WT-MSCs in response to cytokines (Fig. 3B). Using an iNOS inhibitor, L-NMMA, we investigated the potential link between the inhibition of T-cell proliferation and the production of NO. As expected, the production of NO was decreased, and both WT and KO-MSCs were unable to inhibit T-cell proliferation in the presence of L-NMMA (Fig. S1). It suggests that the partial inhibition of T-cell proliferation is caused by a reduction in NO production in KO-MSC.

iNOS is an enzyme responsible for the production of NO in MSCs. Although iNOS mRNA expression was similar in both WT and KO-MSCs, iNOS protein expression was significantly reduced in KO-MSCs (Fig. 3C, D). Since STAT1 and glucose metabolism are involved in the immunomodulation of MSCs, we measured the expression of p-STAT1, STAT1, and glucose transporters [25, 26]. Similar expression levels of p-STAT1 and STAT1 were observed in both MSCs (Fig. 3E). Expression of GLUT1 and GLUT3 was induced by cytokine stimulation. Although the expression levels of GLUT3 are similar in both MSCs, interestingly, the expression of GLUT1 was even higher in KO-MSCs than in WT-MSCs (Fig. S2). Whether the deficiency of *Ahr* affects the expression of GLUT1 will be investigated in the future. These experiments suggested that the reduced iNOS expression in KO-MSCs is likely independent of STAT1 and GLUT expression. The absence of *Ahr* affects iNOS protein expression but not mRNA expression.

### AHR is not involved in iNOS protein stability in MSCs

**Fig. 4:**
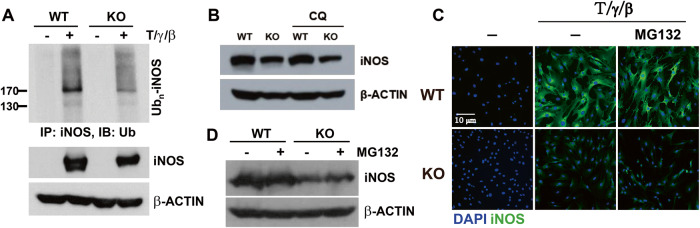
AHR is not involved in iNOS protein stability in MSCs. To measure iNOS protein stability, MSCs were stimulated with TNF-α, IFN-γ, and IL-1β cytokines. **A** The ubiquitination of iNOS was measured using an immunoprecipitation assay. iNOS and β-ACTIN were used for input and loading control. **B** A concentration of 10 μM chloroquine (CQ) was co-treated with three cytokines for 24 h. The levels of iNOS protein were determined using western blotting. **C**, **D** 2 μM MG132 was administered for 3 h one day after cytokine stimulation. iNOS protein levels were determined by immunofluorescence staining and western blotting, respectively. All experiments were performed at least twice, and consistent results were obtained.

To determine the reasons for the reduced levels of iNOS protein in KO-MSCs, we investigated whether AHR affects the stability of the iNOS protein. To investigate the effects of AHR on lysosomal and proteasomal degradation, MSCs were treated with either chloroquine (CQ) or MG132, respectively. iNOS ubiquitination was decreased in KO-MSCs compared to that in WT-MSCs (Fig. 4A). CQ was co-treated with cytokines for 24 h, while MG132 was administrated for 3 h after cytokine stimulation. Neither CQ nor MG132 altered the expression of iNOS protein in KO-MSCs (Fig. 4B–D). These results suggest that AHR does not affect the degradation of iNOS protein in MSCs.

### AHR may control iNOS protein expression by regulating eEF2K expression

**Fig. 5:**
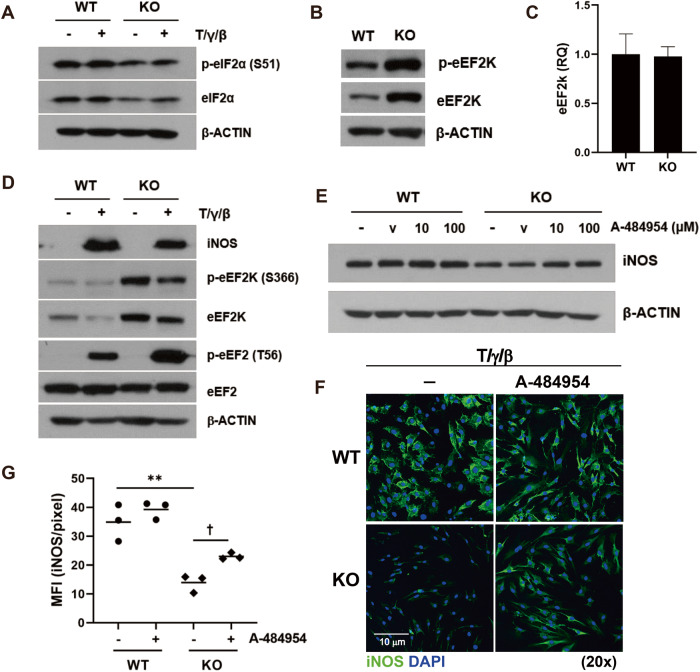
AHR may control iNOS protein expression by regulating eEF2K expression. WT- and KO-MSCs were stimulated with TNF-α, IFN-γ, and IL-1β. **A** Protein synthesis and mRNA translation-related protein levels were measured by western blotting. **B** Protein and (**C**) mRNA levels of eEF2K were measured in unstimulated MSCs by western blotting and qRT-PCR, respectively. **D** eEF2K signaling molecules were observed in cytokine-stimulated MSCs. **E**, **F** MSCs were treated with an eEF2K inhibitor, A-484954, and iNOS protein levels were measured using western blotting and immunofluorescence, respectively. V means DMSO vehicle control. **G** Three different areas in a slide were selected, and the mean fluorescence intensity of iNOS was quantified using ImageJ software. All experiments were performed at least twice, and similar results were obtained. The statistical *p*-value between the comparison of multiple groups was evaluated by a two-way ANOVA followed by Tukey’s multiple comparison test. ^†^*P*-values = ≤ 0.05; *P-values ≤ 0.01 were considered statistically significant.

As observed above, AHR may not directly affect either mRNA transcription or protein degradation of iNOS. It has been reported that UV-induced iNOS expression can be regulated at the translation level by phosphorylation of eukaryotic initiation factor 2 (eIF2α) [27]. The rate of protein synthesis is primarily controlled by translation initiation and elongation [28, 29]. Translation initiation is mostly controlled by eIF2α, and its phosphorylation inhibits protein synthesis [30]. Therefore, we investigated whether AHR is involved in protein synthesis. First, we examined the initiation signaling molecule eIF2α. In KO-MSCs, there was less eIF2α protein and decreased phosphorylation compared to that of the WT (Fig. 5A). We further investigated whether AHR can regulate the translation elongation pathway molecules eEF2K and eukaryotic elongation factor 2 (eEF2). Intriguingly, the level of eEF2K protein and its phosphorylation was significantly increased in KO-MSCs, whereas *Ahr* deficiency did not affect eEF2K mRNA expression (Fig. 5B, C). EEF2K phosphorylates eEF2 on Thr56 (T56), arresting mRNA translation [31–34]. Phosphorylation of eEF2 was also significantly increased in KO-MSCs (Fig. 5D). Congruent with this, in the presence of an eEF2K inhibitor A-484954, the expression of iNOS protein was partially restored in KO-MSCs (Fig. 5E–G). This suggests that the increased eEF2K in MSCs, in the absence of the *Ahr* gene, may be responsible for the decreased expression of the iNOS protein. AHR may be involved in the translation stage by regulating the expression of the eEF2K protein.

### AHR is involved in the ubiquitination and degradation of eEF2K

**Fig. 6:**
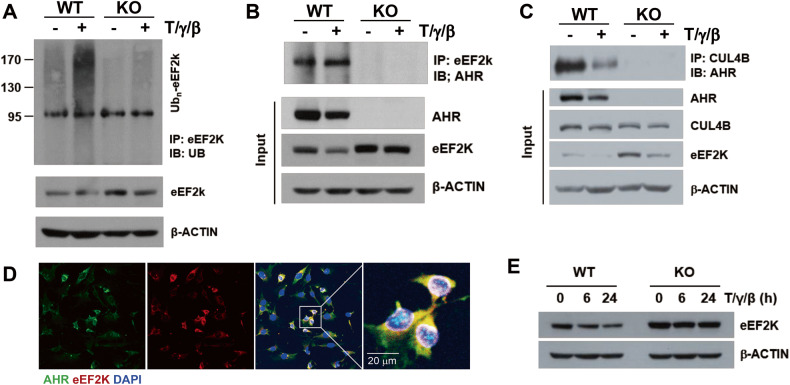
CUL4B^AHR^-mediated eEF2K ubiquitination. **A** MSCs were stimulated with TNF-α, IFN-γ, and IL-1β. EEF2K proteins were immunoprecipitated and detected by an anti-Ub antibody. **B** Protein interaction between eEF2K and AHR was observed using an immunoprecipitation assay. **C** CUL4B proteins were immunoprecipitated, and their interaction with AHR was observed. **D** MSCs were stained with anti-AHR (green) and anti-eEF2K (red) antibodies, and the co-localization was measured by immunofluorescence. DAPI was used to count nuclear staining. **E** After cytokine stimulation, eEF2K protein levels were observed at the indicated time points. β-ACTIN was used as a loading control. All experiments were performed at least twice, and similar results were obtained.

Since AHR acts as an E3 ubiquitin ligase along with CUL4B [14–16], we hypothesized that eEF2K is targeted by the CUL4B^AHR^ E3 ligase. We further hypothesized that deficiency in AHR would lead to decreased ubiquitination of eEF2K, resulting in increased protein levels. The ubiquitination of eEF2K decreased in both basal and cytokine-induced KO-MSCs compared to that in WT-MSCs (Fig. 6A). Co-immunoprecipitation experiments revealed that AHR constitutively interacted with eEF2K and CUL4B, even in the absence of cytokine stimulation (Fig. 6B, C). As shown in immunofluorescence staining, we observed the co-localization of AHR and eEF2K in the cytosol (Fig. 6D). Upon cytokine stimulation, the expression of eEF2K decreased in both WT-MSCs and KO-MSCs, while the ubiquitination of eEF2K increased (Fig. 6A, E*)*. Thus, there might be other ways to ubiquitinate eEF2K, and AHR does not appear to be the only E3 ligase that targets eEF2K. Nevertheless, the expression level of eEF2K is still higher in KO-MSCs than in WT-MSCs.

### AHR antagonist CH223191 can rescue eEF2K degradation in WT-MSCs

**Fig. 7:**
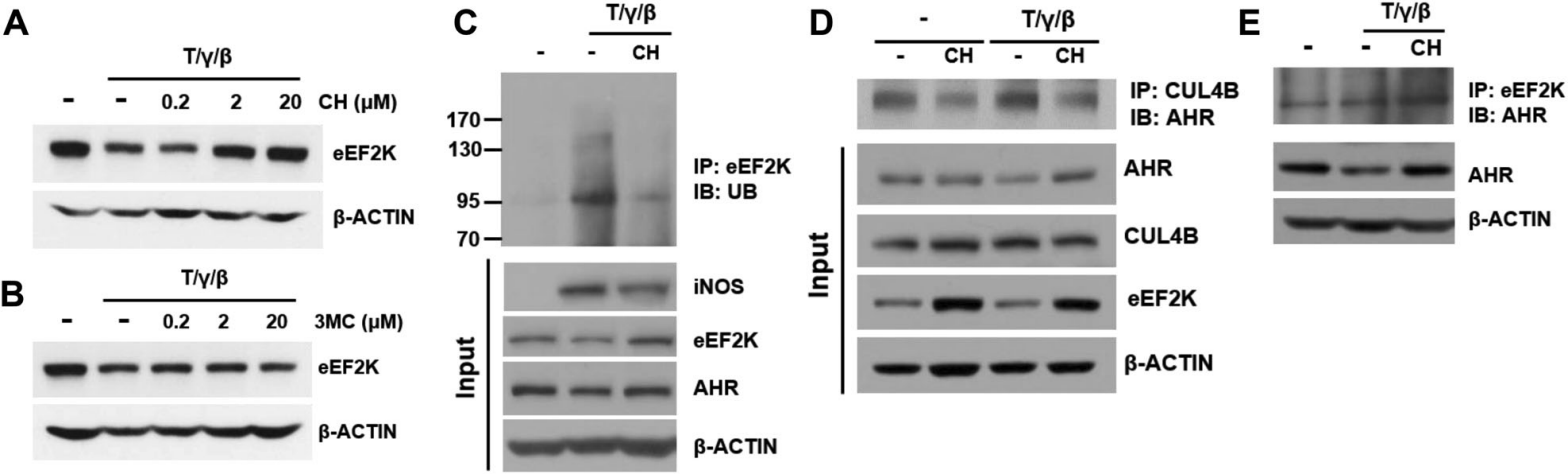
AHR antagonist can rescue eEF2K degradation in WT-MSCs. **A**, **B** Cytokine-stimulated WT-MSCs were treated with different concentrations of CH223191 and 3MC. EEF2K protein levels were measured. **C**–**E** Cytokine-stimulated WT-MSCs were treated with 20 μM CH223191 and eEF2K ubiquitination and interaction of AHR and CUL4B were observed. All experiments were performed at least twice, and similar results were obtained.

**Fig. 8:**
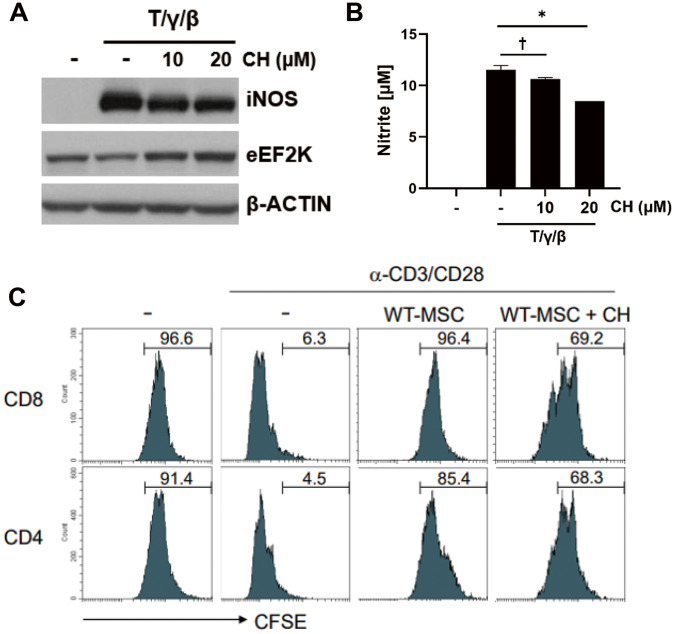
Rescued T-cell proliferation by an AHR antagonist, CH223191. **A**, **B** WT-MSCs were stimulated by cytokines in the presence of CH223191. iNOS and eEF2K protein expression and NO production were measured by western blotting and ELISA, respectively. **C** Isolated splenocytes from WT mice were stimulated with anti-CD3 and anti-CD28 antibodies in the presence of WT-MSCs and CH223191. CFSE-stained cell divisions were analyzed by flow cytometry. All experiments were performed at least twice, and similar results were obtained. ƚ*p* < 0.05, *p < 0.01 significantly different from the positive control bar. The statistical *p*-value between the comparison of multiple groups was evaluated by a two-way ANOVA followed by Tukey’s multiple comparison test. ^†^*P*-values ≤ 0.05; *P-values ≤ 0.01 were considered statistically significant.

It has been reported that the E3 ligase function of AHR is ligand-dependent [15]. Therefore, we investigated whether AHR ligands are required for the ubiquitination and degradation of eEF2K. First, we investigated whether the AHR antagonist CH223191 and agonist 3MC can affect the expression of the eEF2K protein. Cytokine-induced degradation of eEF2K was rescued in a dose-dependent manner by CH223191, while 3MC did not affect the expression of eEF2K (Fig. 7A, B). It seems that additional exogenous AHR agonists are probably not required for the degradation of eEF2K. Consistent with the increased expression of eEF2K by CH223191, the ubiquitination of eEF2K was inhibited, likely due to the disruption of the CUL4B interaction (Fig. 7C, D). Because CH223191 did not affect the interaction between AHR and eEF2K (Fig. 7E). The interaction between AHR and CUL4B is required for the degradation of eEF2K. The AHR likely regulates the basal level of eEF2K protein expression. We further evaluated whether CH223191 treatment affects the expression of iNOS protein and the production of NO. In the presence of CH223191, iNOS protein expression and NO production decreased in a dose-dependent manner (Fig. 8A, B). This was associated with an increase in T-cell proliferation when WT-MSCs were co-cultured with T cells in the presence of CH223191 (Fig. 8C). This suggests that the AHR in MSCs may function as an immunomodulatory factor that regulates the ubiquitination of eEF2K, thereby controlling the synthesis of iNOS protein and its resulting levels of NO.

## Discussion

The immunomodulatory properties of MSCs manifest in the suppression of a wide range of innate and adaptive immune cells, including lymphocytes, natural killer cells, macrophages, and dendritic cells. In mouse MSCs, iNOS produces NO, a powerful inhibitory molecule in T cells [5]. NO arrests the cell cycle by inhibiting STAT5 [9]. In a previous study, we demonstrated that NO produced by MSCs inhibits CD25 translation in T cells by regulating the LKB1-AMPK-mTOR pathway, thereby suppressing T cells [24]. It is well known that iNOS is induced by inflammatory cytokines such as IFN-γ, TNF-α, and IL-1β. Cytokine-mediated activation of STAT1 and NF-κB has been well-studied for the induction of iNOS [35]. In addition, TCDD-induced AHR activation has been reported to upregulate iNOS expression and increase NO production in both MSCs and endothelial cells, thereby enhancing their immunosuppressive ability [21, 36]. Several studies have reported that the ligand-activated AHR plays a role in the anti-inflammatory and immunomodulatory effects of MSCs [20, 21]. However, the exact mechanism by which AHR induces iNOS has not been well elucidated. We isolated MSCs from *Ahr*−/− mice and compared their immunomodulatory functions with those of WT-MSCs in the treatment of GVHD. We observed a decrease in the therapeutic efficacy of KO-MSCs compared to WT-MSCs. This decrease is likely attributed to a reduction in their inhibitory function on T cells, as evidenced by the decreased expression of iNOS and NO. Our data further emphasize the role of AHR in regulating eEF2K through the CUL4B^AHR^-E3 ligase complex, which may control iNOS translation. This highlights a previously neglected pathway for AHR-mediated changes in protein expression that can either contrast with or modify its function as a transcription factor. Several reports have shown that AHR-mediated iNOS induction occurs. It has also been demonstrated that AHR agonists, such as benzo[a]pyrene and TCDD, induce NO production by increasing iNOS protein levels [21, 36–38]. *Ahr*-null macrophages exhibit decreased NO production and polarize towards the M2 phenotype [39]. A putative XRE sequence, 5’-CACGC-3’ (−788 to −315), has been suggested in the promoter region of the mouse iNOS gene. Additionally, the involvement of AHR in LPS-induced iNOS gene transcription has been proposed in microglia [40]. Wheeler et al. showed that the conditional deletion of *Ahr* in endothelial cells inhibits the upregulation of iNOS protein expression induced by TCDD treatment during influenza virus infection. However, the conditional deletion of *Ahr* in epithelial cells did not have the same effect. It seems that the functions of AHR in terms of iNOS induction may be specific to certain cell types [36]. Using the DNA-binding domain mutated *Ahr*^*dbd/dbd*^ mice, they showed that the DNA-binding domain of the AHR protein is required for inducing iNOS in the lung after TCDD treatment during influenza virus infection. However, they did not observe ligand-activated AHR binding to the putative XREs in the iNOS promoter [36]. It seems that the DNA-binding domain functions not only for transcriptional activity but also for other purposes. Dou et al. identified PPARγ as a target of CUL4B^AHR^-E3 ligase [17]. They showed that PPARγ binds to AHR mainly through its DNA-binding domain. Based on these reports, we can speculate that the DNA-binding domain of AHR may be necessary for binding E3 ligase targets.

Here, we observed decreased iNOS protein expression, but not iNOS mRNA expression, in *Ahr* KO-MSCs stimulated by inflammatory cytokines. Instead, we observed increased eEF2K protein expression in KO-MSCs, which acts as a negative regulator of protein synthesis, translation, and cell growth. EEF2k phosphorylates eEF2 at the Thr56 site and impairs its binding to the ribosome, thus slowing down the elongation stage of protein synthesis [41–44]. The eEF2K protein is degraded in response to stimuli (nerve growth factor, insulin-like growth factor-1) via a proteasome-dependent pathway, in which a ubiquitin E3 ligase ß-TrCP is required [45, 46]. Here, we observed decreased eEF2K protein expression and increased ubiquitination following cytokine stimulation in both WT-MSCs and KO-MSCs. One possibility is that the SCF^β-TrCP^ ubiquitin E3 ligase regulates the stability of the eEF2K protein [45–47]. Nevertheless, AHR has also been involved in eEF2K ubiquitination, as the expression level of eEF2K in KO-MSCs remains higher than that in WT-MSCs after cytokine stimulation. Upon treatment with the eEF2K inhibitor, A484954, iNOS protein levels were partially rescued in KO-MSCs. This suggests that increased levels of eEF2K in KO-MSCs are involved in the inhibition of iNOS protein expression. In addition, we observed a constitutive interaction between eEF2K and AHR, as well as a decrease in the ubiquitination of eEF2K in KO-MSCs. Since the CUL4B^AHR^-E3 ligase might be involved in the ubiquitination and degradation of eEF2K, the AHR may contribute to the cell-specific regulation of protein expression in response to external stimuli.

The CUL4B^AHR^-E3 ligase complex requires an AHR ligand for complex assembly and catalytic activity [48]. The AHR antagonist, CH223191, rescued cytokine-inhibited eEF2K expression in a dose-dependent manner, but not 3MC. In addition, the binding of AHR to CUL4B was inhibited by CH223191, and the ubiquitination of eEF2K was decreased. Therefore, CUL4B is required for AHR-mediated eEF2K degradation. Indeed, AHR constitutively controls the degradation of eEF2K, even without cytokine stimulation. Furthermore, we observed the co-localization of AHR with Erp72, an ER marker that co-localized with eEF2K in the cytosol (Fig. S3A, B). We believe that endogenous ligands-activated AHR in the cytosol may control the steady-state level of eEF2K protein, which controls the fine-tuning of the translational machinery in MSCs. Future studies should investigate the precise roles of ligands in AHR E3 ligase activity.

In summary, we have demonstrated that AHR functions as an E3 ligase together with CUL4B to degrade eEF2K. This process regulates the expression of the iNOS protein, which is involved in the immunosuppressive function of MSCs. In addition, eEF2K plays a significant role in several diseases, including neurodegenerative diseases, cancer, cardiovascular diseases, muscular hypertrophy, and other immune pathologies [44]. The AHR-eEF2K mechanism may help us understand disease pathologies and develop therapeutic drugs.

## Materials and methods

### Mice

The bone marrows of *Ahr* + /+ and *Ahr*−/− littermate mice were obtained from mice bred at the Leibniz-Research Institute for Environmental Medicine (Düsseldorf, Germany) (Strain B6.129AHR^tm1Bra^). C57BL/6 and BALB/c mice (Orient Co, Seongnam, South Korea) were used for the T cells and GVHD experiments. The mice were housed in a specific pathogen-free barrier facility at Inha University. All of the animal experiments were approved by the Institutional Animal Care and Use Committee (INHA 181212-607-5).

### Genotyping

Using phenol extraction, genomic DNA was isolated from *Ahr* wild-type (WT)- and knockout (KO)-MSCs. *Ahr* gene deficiency was confirmed by genomic PCR. The *Ahr* gene was amplified using specific primers (Jackson Laboratory, Bar Harbor, ME, USA): oIMR0443, 5’-GGA TTT GAC TTA ATT CCT TCA GCG G-3’; oIMR0444: 5’-TCT TGG GCT CGA TCT TGT GTC AGG AAC AGG-3’; and oIMR8162: 5’-TGG ATG TGG AAT GTG TGC GAG-3’. The oIMR0443 and oIMR0444 pairs were used for the WT gene, and the oIMR0444 and oIMR8162 pairs were used to detect the KO gene (Fig. S4A). The conditions for PCR running were as follows: after 2 min at 94 °C, 10 cycles were repeated with 20 s at 94 °C, 15 s at 65 °C, and 10 s at 68 °C. Then, 28 cycles were repeated with 15 s at 94 °C, 15 s at 60 °C, 10 s at 72 °C, 2 min at 72 °C, and then held at 10 °C.

### Antibodies and chemicals

Listed in supplementary information Tables 1 and 2.

### MSC culture

MSCs were isolated from the bone marrow of *Ahr* + */+* and *Ahr−/−* C57BL/6 littermates. Cells were isolated from humeri, tibiae, and femurs and incubated in a 100 mm dish with Dulbecco’s modified Eagle medium (DMEM) with a low glucose concentration (Gibco, Thermo Fisher Scientific, MA, USA), 10% fetal bovine serum (FBS) (Gibco, Australia), and 1% antibiotic-antimycotic (AA) (Gibco) at 37 °C, with 5% CO_2_. After 1 day, non-adherent cells were removed, and adherent cells were incubated with new media until 70% confluence. Cells from passages 10 to 18 were used in this study. MSCs were regularly tested for mycoplasma contamination. Under stimulation conditions, MSCs were treated with 10 ng/mL TNF-α, 20 ng/mL IFN-γ, and 10 ng/mL IL-1β for 24 h or the indicated time points.

### Characterization of MSCs

MSCs were characterized according to cell surface markers, such as positive for CD44, CD105, and Sca-1, and negative for CD14, CD34, and MHC class II (Fig. S4B). All of the antibodies were purchased from BD Biosciences (Franklin Lakes, NJ, USA).

### Differentiation

Twenty-four hours before differentiation, 5 × 10^4^ MSCs/well were seeded into a four-well plate. The adipogenic differentiation medium (high glucose-DMEM, 1% AA, 10% newborn calf serum (NCS), 10^−7^ M Dexamethasone, 100 μM Indomethacin, 0.5 M IBMX, 10 μg/mL Insulin) was changed 3 times in a 1-week. MSCs were cultured for 14 days and stained with Oil Red O solution (0.5% Oil Red O in isopropanol) purchased from Sigma-Aldrich (Burlington, MA, USA). The osteogenic differentiation medium (α-MEM media, 1% AA, 10% FBS, 50 μg/mL ascorbic acid, 10 mM β-glycerophosphate, 1 mM dibutyryl-cAMP, 10^−8^ M dexamethasone) was changed 3 times at 1-week. MSCs were cultured for 21 days and stained with Alizarin Red S solution (1% Alizarin Red in dH_2_O) purchased from Sigma-Aldrich. Twenty-four hours before chondrogenic differentiation, 5 × 10^5^ MSCs/tube were seeded in a 15 mL conical tube. The cells underwent centrifugation at 2000g for 15 min. The chondrogenic differentiation media (α-MEM media, 1% AA, 50 μg/mL ascorbic acid, 10^−7^ M dexamethasone, 10 ng/mL TGF-β1, TGF-β3, 40 μg/mL proline, ITS premix) were changed 3 times at 1-week. MSCs were cultured for 21 days, and the cell pellets were embedded in an OCT compound, rapidly frozen in liquid nitrogen, and subsequently stored at −80 °C. Sections were cut into 8-μm slices and stained with a Safranin O solution (0.5% Safranin O in dH_2_O) purchased from Sigma-Aldrich (Fig. S4C).

### Co-culture of lymphocytes with MSCs

Primary lymphocytes from the mouse spleen and lymph nodes were cultured with MSCs at a 20:1 ratio. MSCs (5 × 10^4^) were co-cultured with 1 × 10^6^ lymphocytes and stimulated with 1 μg/mL anti-CD3 (145-2C11) and anti-CD28 (37.51) mouse antibodies (mAb) (Biogems, Westlake Village, CA, USA) in 1 mL of RPMI-1640 (Hyclone, GE Healthcare Life Science, PA, USA) in a 24-well plate. RPMI-1640 was used as the culture medium, containing 10% FBS, 1% AA, and 0.1% 2-mercaptoethanol (Gibco). Cells were incubated for 24, 48, and 72 h at 37 °C, with 5% CO_2_.

### Flow cytometry

After co-culture, lymphocytes were stained with the appropriate mixture of mAbs diluted in FACS buffer (PBS with 1% BSA and 20 nM EDTA) at 4 °C for 20 min, then washed once with FACS buffer and fixed in 4% PFA (paraformaldehyde; Biosesang, Seongnam, South Korea). Intracellular proteins were stained using the Cytofix/Cytoperm^TM^ Fixation/Permeabilization Kit according to the manufacturer’s instructions (BD Biosciences). Cells were then examined using the BD FACS Verse™ instrument (BD Biosciences), and the data were analyzed using FlowJo software (BD Biosciences). The FACS antibodies used were CD4 (RM4-5, GK1.5), CD8 (53-6.7), CD25 (3C7), and CD122 (TM-β1), purchased from BD Biosciences and Invitrogen (Carlsbad, CA, USA).

### Immunofluorescence assay

MSCs (5 × 10^4^) were cultured on a coverslip in a 24-well plate with 1 mL of DMEM. After overnight incubation, the cells were treated with 10 ng/mL TNF-α, 20 ng/mL IFN-γ, and 10 ng/mL IL-1β for 24 and 48 h. The cells were then washed with PBS and fixed with 100% methanol for 3 min, followed by three time washes with PBS. Then, the cells were incubated overnight with primary anti-iNOS, anti-AHR, anti-ERp72, and anti-eEF2K antibodies at 4 °C and then incubated with rabbit anti-mouse IgG-Alexa Fluor 488 (A11008, Invitrogen, Carlsbad, CA, USA) or goat anti-rabbit IgG-Alexa Fluor 594 (A11012, Invitrogen) secondary antibodies for 2 h at room temperature in a dark place. As a negative control, 2^nd^ antibody was stained alone (Fig. S5). Nuclei were stained with DAPI (Molecular Probes, Invitrogen). Cells were mounted and observed under a confocal laser microscope (Olympus Corp., Tokyo, Japan).

### Analysis of T-cell division

Lymphocytes were washed twice with DPBS. The cells (5 × 10^7^) were suspended in 500 μL of DPBS. We then added 1:1,000 dilutions of 1 mM carboxyfluorescein diacetate succinimidyl ester (CFSE; Invitrogen) and incubated them for 15 min at 37 °C. To stop the reaction, 10 mL of cold DPBS was added, and the cells were washed twice. The stained lymphocytes were suspended in the RPMI culture medium. MSCs (5 × 10^4^) were co-cultured with 1 × 10^6^ CFSE-stained lymphocytes and stimulated with 1 μg/mL anti-CD3 and anti-CD28 mAb in 1 mL of RPMI-1640 on a 24-well plate. After 48 and 72 h, the cells were stained with CD4 and CD8 antibodies (BD Biosciences), and T-cell divisions were analyzed by flow cytometry. For iNOS inhibition, 1 mM N^G^-Methyl-L-arginine acetate salt (L-NMMA; Sigma-Aldrich) was co-treated with anti-CD3 and anti-CD28 mAb.

### ELISA

The levels of IL-2, IL-17A, and IFN-γ in the culture medium derived from the co-culture media were measured by ELISA, according to the manufacturer’s protocol (BD Biosciences). The results were measured using an ELISA reader at a wavelength of 450 nm.

### Nitric oxide (NO) assay

MSCs (5 × 10^4^) were cultured with 10 ng/mL TNF-α (R&D Systems, Minneapolis, MN, USA), 20 ng/mL IFN-γ (BD Biosciences), and 10 ng/mL IL-1β (PeproTech, Cranbury, NJ, USA) in 1 mL DMEM in a 24-well plate. After 24, 48, and 72 h, conditioned media were used for the NO assay. NO was measured using a Griess reagent (Sigma-Aldrich). The conditioned medium (100 μL) and nitrite standard (100 μL) were mixed with 100 μL Griess reagent. The absorbance was measured at 540 nm using an ELISA reader. The NO concentration was calculated using a NaNO_2_ standard reference curve.

### Quantitative real-time reverse transcription polymerase chain reaction (qRT-PCR)

MSCs (4 × 10^5^) were cultured in a 100 mm cell culture dish with 10 mL DMEM overnight and then treated with 10 ng/mL TNF-α, 20 ng/mL IFN-γ, and 10 ng/mL IL-1β. After 24 h, MSCs were harvested to extract the total mRNA. mRNA was extracted from MSCs using the TRIzol reagent (Gibco). According to the manufacturer’s instructions, cDNA was synthesized using an AMV reverse transcriptase Kit (Promega, Madison, WI, USA). The cDNA products were amplified using the Kapa SYBR qPCR Kit master mix (2x) from Kapa Biosystems (Sigma-Aldrich). cDNA was amplified using primers specific for iNOS and β-actin (Qiagen, Hilden, Germany). The reactions were carried out on a StepOnePlus Real-Time PCR system (Applied Biosystems, Waltham, MA, USA). The conditions for PCR were as follows: for activating the DNA polymerase, a hot start was performed for 10 min at 95 °C, then cycling at 95 °C for 15 s, and 60 °C for 1 min for a total of 40 cycles.

### Western blot analysis

MSCs (4 × 10^5^) were cultured in a 100 mm cell culture dish with 10 mL of DMEM. After an overnight incubation, the cells were treated with 10 ng/mL TNF-α, 20 ng/mL IFN-γ, and 10 ng/mL IL-1β for 24 h or indicated time points. Treated cells were harvested and lysed in RIPA buffer after being washed twice with cold PBS. Lysates were evaluated using standard western blotting methods. Proteins on the PVDF membrane were probed with the indicated primary antibodies. The secondary antibodies used were horseradish peroxidase-conjugated anti-rabbit (Invitrogen, Carlsbad, CA, USA) or anti-mouse IgG (Invitrogen). All original western blot data are shown in the supplemental material.

### Immunoprecipitation assay

MSCs (4 × 10^5^) were cultured in a 100 mm cell culture dish. Under stimulation conditions, MSCs were treated with 10 ng/mL TNF-α, 20 ng/mL IFN-γ, and 10 ng/mL IL-1β for 24 h or the indicated time points. The cells were then washed with ice-cold PBS and lysed with immunoprecipitation (IP) buffer (10 mM Tris-HCl, pH 7.5, 2 mM EDTA, 0.5% NP-40, and 150 mM NaCl, supplemented with protease and phosphate inhibitor cocktail). The lysates were incubated with anti-iNOS, anti-CUL4B, or anti-eEF2K antibodies for 1 h at 4 °C. Protein-G Sepharose beads (25 μl) were added to each IP tube and incubated at 4 °C for 1 h overnight. The supernatants were removed, and the beads were washed three times with IP buffer. Finally, the immunoprecipitated proteins were eluted by heating at 95 °C for 5 min, and the IP products were detected by western blotting, as described in the figures.

### Graft versus host disease (GVHD) model

Recipient BALB/c mice (female, 10 weeks old, H2d) were irradiated with a dose of 7.0 Gy using CLINIC iX medical linear accelerators (Varian, Palo Alto, CA, USA). 24 h after irradiation, 5 × 10^6^/100 μL of bone marrow cells and 5 × 10^6^/100 μL of splenocytes (isolated from C57BL/6 mice (female, 6 weeks old, H2b)) were injected intravenously into the lateral tail vein. After inducing GVHD, on days 1, 3, and 5 after transplantation, 2 × 10^5^ WT- and KO-MSCs were injected intravenously to observe the therapeutic efficacy of the survival rate, body weight, and clinical scores. Clinical symptoms were scored as shown in supplemental table 3. This study was conducted using four animal groups: bone marrow was injected as a negative control (naïve group: *N* = 5), a control group where GVHD was induced and a cell vehicle was administered (PBS group: *N* = 10), experimental groups, where GVHD was induced and *Ahr* WT- (*N* = 10) and *Ahr* KO-MSCs (*N* = 10) were administered intravenously.

### Statistical analysis

All data analyses were performed using GraphPad Prism version 9.5.1, GraphPad Software. The statistical *p*-value between the comparison of multiple groups was evaluated by a two-way ANOVA followed by Tukey’s multiple comparison test. ^†^*P*-values ≤ 0.05; *P-values ≤ 0.01; **P-values ≤ 0.001 were considered statistically significant. Specific tests used are stated in the figure legends.

### Supplementary information

Supplementary information

Original Data File

## Data Availability

All study data is included in the articles and/or supplemental figures and tables.
