# Supplementary material for: The aryl hydrocarbon receptor controls mesenchymal stromal cell-mediated immunomodulation via ubiquitination of eukaryotic elongation factor-2 kinase
Source: Cell Death Dis. 2023 Dec 9;14(12):812. doi: 10.1038/s41419-023-06341-7 (PMC10710493; doi:10.1038/s41419-023-06341-7)
Supplement: Supplementary file 1 — Supplementary information [file 41419_2023_6341_MOESM1_ESM.pdf]

# The aryl hydrocarbon receptor controls mesenchymal stromal cell-mediated immunomodulation via ubiquitination of eukaryotic elongation factor-2 kinase

Enkhmaa Lkhagva-Yondon[1,3,7], Myeong Seong Seo[1,3,7], Yena Oh[1,2], Jong Hun Jung[1,3], Eunhae Jeon[1,2], Kwangmin Na[1], Hyun Seung Yoo[1,2], Woo Chul Kim[4], Charlotte Esser[5], Sun U Song[1,2,6], and Myung-Shin Jeon[1,2,3,6]

[1] Translational Research Center, Inha University Hospital, Incheon 22332, Republic of Korea

[2] Department of Molecular Biomedicine, College of Medicine Inha University, Incheon 22212, Republic of Korea

[3] Program in Biomedical Science & Engineering Inha University, Incheon 22212, Republic of Korea

[4] Department of Radiation Oncology, Inha University Hospital, Incheon 22332, Republic of Korea

[5] IUF-Leibniz Research Institute for Environmental Medicine, Düsseldorf 40021, Germany

[6] SCM Lifescience, Incheon 21999, Republic of Korea

Correspondence: Prof. Myung-Shin Jeon, Translational Research Center, Inha University Hospital, College of Medicine, B/306 Jungseok Bldg., 366 Seohae-daero, Jung-Gu, Incheon 22332, Korea. (Tel.) +82-32-890-3682; (Fax) +82-32-890-2462; (E-mail) msjeon@inha.ac.kr

[7] These authors contributed equally to this work.

**Supplement Figure 1**

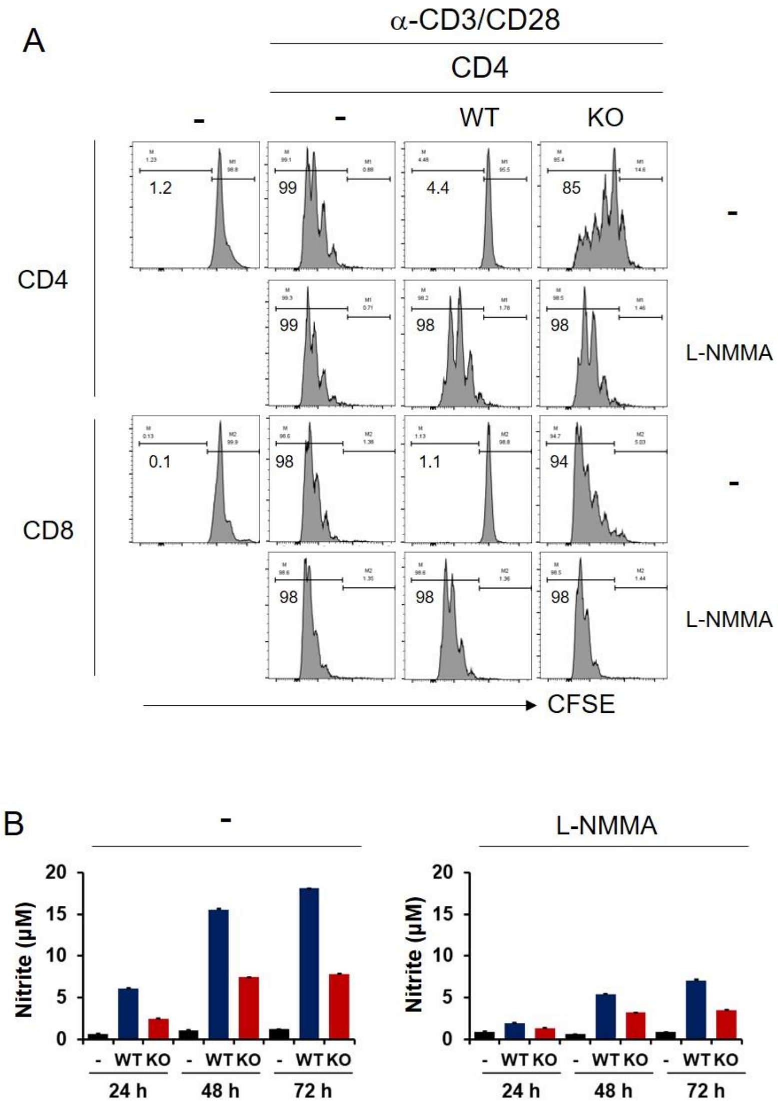


**Fig. 1 Effect of *Ahr*$^{-/-}$ MSC on T cell proliferation in the presence of iNOS inhibitor**

Lymphocytes were stained with CFSE and stimulated with 1 μg/mL anti-CD3 and anti-CD28 Abs in the presence of 1 mM L-NMMA, an iNOS inhibitor. (A) After 72 h, T-cell divisions were analyzed by flow cytometry. (B) NO production was measured in the culture media using ELISA.

## Supplement Figure 2

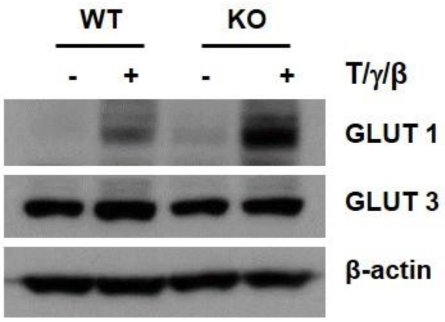


**Fig. 2 Effect of *Ahr* deficiency on glucose transporters**

WT- and KO-MSCs were stimulated by TNF-α, IFN-γ, and IL-1β. After 24 h, the expression of GLUT1 and GLUT3 proteins was measured by western blotting.

## Supplement Figure 2

A

B

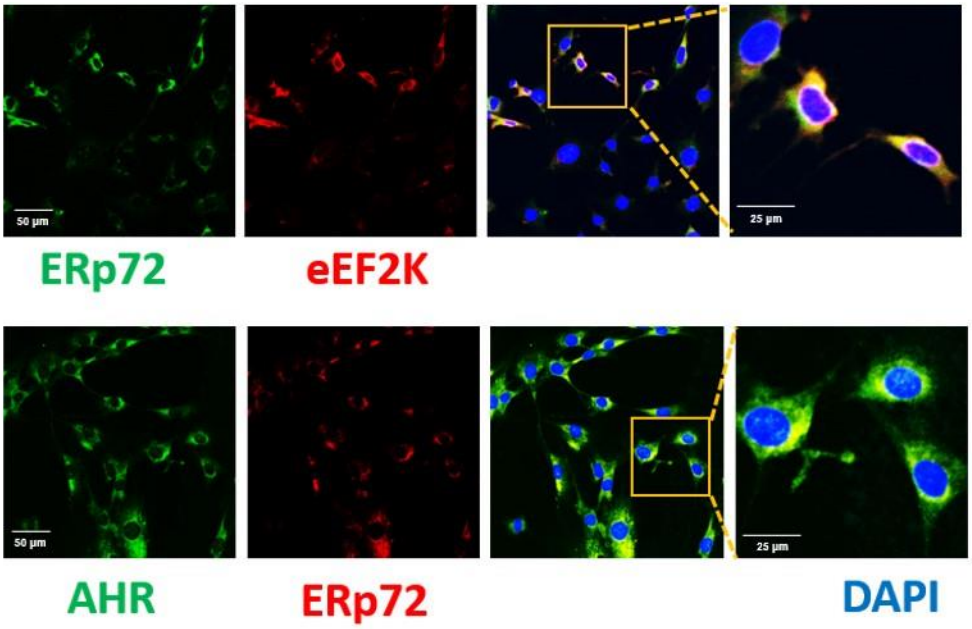


**Fig. 3 Co-localization of AHR and eEF2K with ER marker ERp72**

(A) MSCs were stained with anti-AHR (green) and anti-eEF2K (red) antibodies and co-localization was measured by immunofluorescence. (B) MSCs were stained with anti-AHR (green) and anti-ERp72 (red) antibodies and immunofluorescence measured co-localization. DAPI was used to count nuclear staining. All experiments were repeated at least twice, and similar results were obtained.

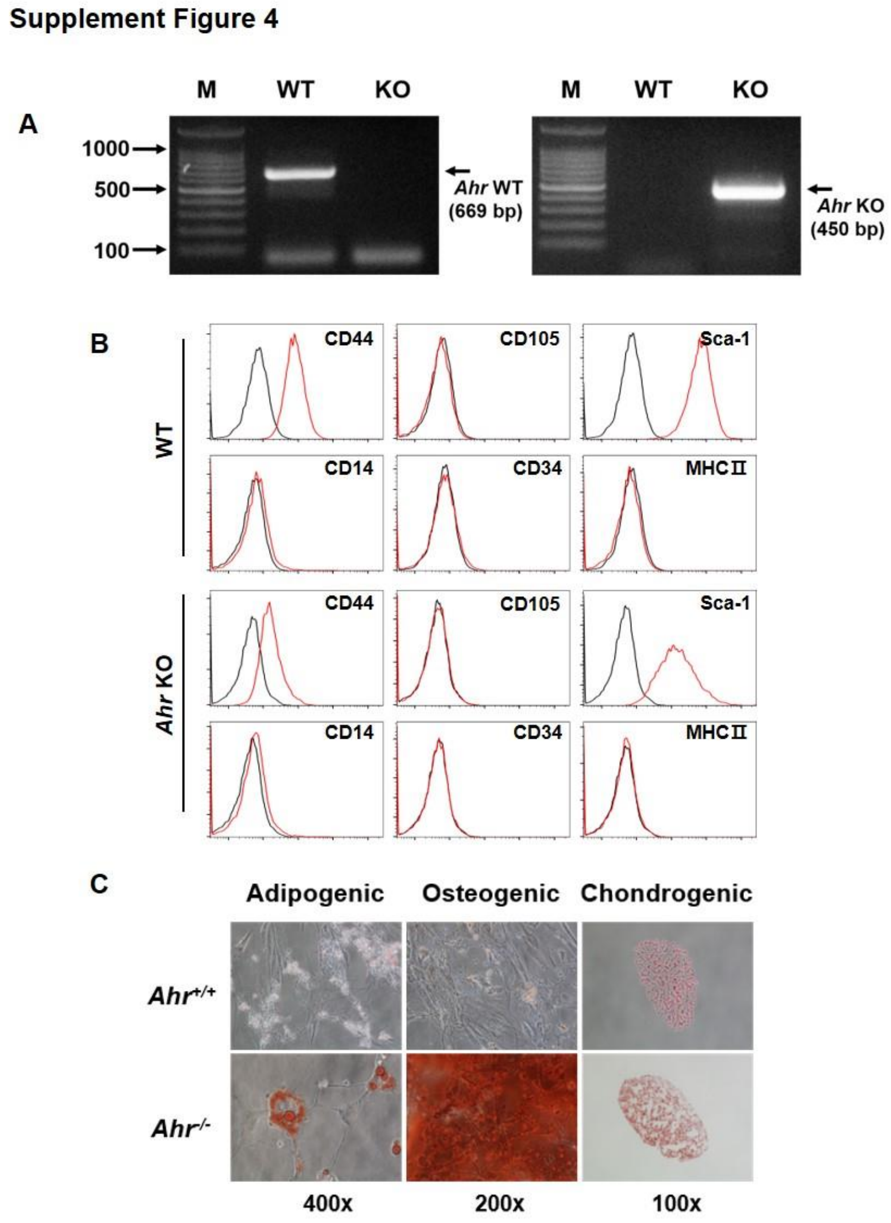


**Fig. 4 Genotyping, characterization, and differentiation of MSCs**

(A) Genotyping of *Ahr*. Using phenol extraction, genomic DNA was isolated from *Ahr* wild-type (WT)- and knockout (KO)-MSCs. *Ahr* gene deficiency was confirmed by genomic PCR. (B) MSCs were characterized according to cell surface markers, such as positive for CD44, CD105, and Sca-1 and negative for CD14, CD34, and MHC class II. (C) Three lineage differentiation of MSCs was performed. All experiments were repeated at least twice, and similar results were obtained.

## Supplement Figure 5

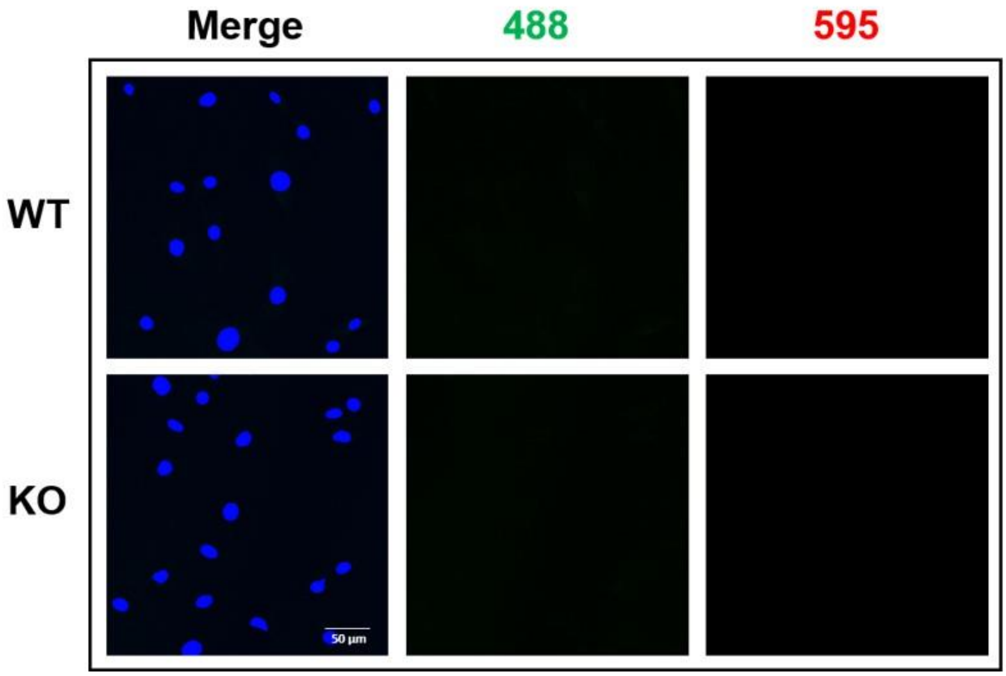


**Fig. 5 Immunofluorescence staining control**

2nd Antibodies were stained alone as a negative control.

**Supplemental Table 1 Antibodies**

| Antibody | Source | Identifier | Application |
|---|---|---|---|
| Actin | Santa Cruz ( CA, USA) | #47778 | WB (1/1,000) |
| AhR | Proteintech (Rosemont, IL, USA) | #17840-I-AP | WB (1/1,000), IF (1/100) |
| b-TRCP | Cell Signaling (Boston, MA, USA) | #11984S | WB (1/1,000) |
| CD14 | BD Biosciences ( NJ, USA) | #560638 | FC (1/100) |
| CD34 | Biolegend (CA, USA) | #128611 | FC (1/100) |
| CD44 | BD Biosciences ( NJ, USA) | #553133 | FC (1/100) |
| CD105 | Biolegend (CA, USA) | #120407 | FC (1/100) |
| CUL4B | Proteintech (Rosemont, IL, USA) | #12916-1-AP | WB (1/1,000), IP (4 μg) |
| eEF2 | Cell Signaling (Boston, MA, USA) | #2332S | WB (1/1,000) |
| eEF2k | Santa Cruz ( CA, USA) | #390710 | WB (1/500), IP (4 μg), IF (1/100) |
| eIF2a | Cell Signaling (Boston, MA, USA) | #9722S | WB (1/1,000) |
| ERp72 | Cell Signaling (Boston, MA, USA) | #5033S | IF 1/100 |
| GLUT1 | Abcam (Cambridge, UK) | #115730 | WB (1/1,000) |
| GLUT3 | Proteintech (Rosemont, IL, USA) | #20403-1-AP | WB (1/1,000) |
| INOS | Cell Signaling (Boston, MA, USA) | #13120 | WB (1/2000), IP (4 μg) |
| MHC II | BD Biosciences (NJ, USA) | #553623 | FC (1/100) |
| p-eEF2 (Thr56) | Cell Signaling (Boston, MA, USA) | #2331S | WB (1/1,000) |
| p-eEF2k (S366) | Cell Signaling (Boston, MA, USA) | #3691 | WB (1/1,000) |
| p-eIF2a (S51) | Cell Signaling (Boston, MA, USA) | #3597S | WB (1/1,000) |
| STAT1 | Cell Signaling (Boston, MA, USA) | #14944 | WB (1/1,000) |
| p-STAT1 (S727) | Cell Signaling (Boston, MA, USA) | #8826 | WB (1/1,000) |
| Ubiquitin | Santa Cruz ( CA, USA) | #8017 | WB (1/1,000) |
| Mouse IgG1-HRP | Cell Signaling (Boston, MA, USA) | #7076S | WB (1/1,0000) |
| Rabbit Ig-G1-HRP | Invitrogen (Carlsbad, CA, USA) | #31460 | WB (1/1,0000) |

**Supplemental Table 2. Chemical lists**

| Name | Source | Identifier |
|---|---|---|
| A-484954 | Sigma (St Louis, MO, USA) | #324516 |
| Choloroquine diphosphate salt | Sigma (St Louis, MO, USA) | #C6628 |
| CH-223191 | Sigma (St Louis, MO, USA) | #C8124 |
| Cycloheximide | Sigma (St Louis, MO, USA) | #C7698 |
| 6-formylindolo [3, 2-b] carbazole | Sigma (St Louis, MO, USA) | #SML1489 |
| L-NMMA | Sigma (St Louis, MO, USA) | #53308-83-1 |
| MG132 | Sigma (St Louis, MO, USA) | #474790 |
| 3-Methylcholanthrene | Sigma (St Louis, MO, USA) | #442388 |

**Adipogenic differentiation**

| Name | Source | Identifier |
|---|---|---|
| Dexamethasone | Sigma (St Louis, MO, USA) | #D8893 |
| Indomethacin | Sigma (St Louis, MO, USA) | #17378 |
| Insulin | Sigma (St Louis, MO, USA) | #19278 |
| Isobutylmethylxanthine | Sigma (St Louis, MO, USA) | #17018 |

**Chondrogenic differentiation**

| Name | Source | Identifier |
|---|---|---|
| Ascorbic acid | Sigma (St Louis, MO, USA) | #A4403 |
| Dexamethasone | Sigma (St Louis, MO, USA) | #D8893 |
| ITS Premix | BD Bioscience | #354352 |
| L-Proline | Sigma (St Louis, MO, USA) | #P5607 |
| TGF-β1 | R&D Systems | #240-B |
| TGF-β3 | R&D Systems | #243-B |

**Osteogenic differentiation**

| Name | Source | Identifier |
|---|---|---|
| Ascorbic acid | Sigma (St Louis, MO, USA) | #A4403 |
| β-glycerolphosphate | Sigma (St Louis, MO, USA) | #G9422 |
| Dexamethasone | Sigma (St Louis, MO, USA) | #D8893 |
| dibutyryl cyclin AMP | Sigma (St Louis, MO, USA) | #D067 |

**Supplemental Table 3. GVHD Clinical scores**

| | Severity scores | | |
|---|---|---|---|
| Symptoms | normal | mild | severe |
| Ruffled hair | 0 | 1 | 2 |
| Hunched back | 0 | 1 | 2 |
| Diarrhea | 0 | 1 | 2 |
| Damaged skin | 0 | 1 | 2 |
| Ascites | 0 | 1 | 2 |
| Total | | | 10 |
| Dead | | | 10 |
