# Supplementary material for: The aryl hydrocarbon receptor controls mesenchymal stromal cell-mediated immunomodulation via ubiquitination of eukaryotic elongation factor-2 kinase
Source: Cell Death Dis. 2023 Dec 9;14(12):812. doi: 10.1038/s41419-023-06341-7 (PMC10710493; doi:10.1038/s41419-023-06341-7)

Fig. 3D

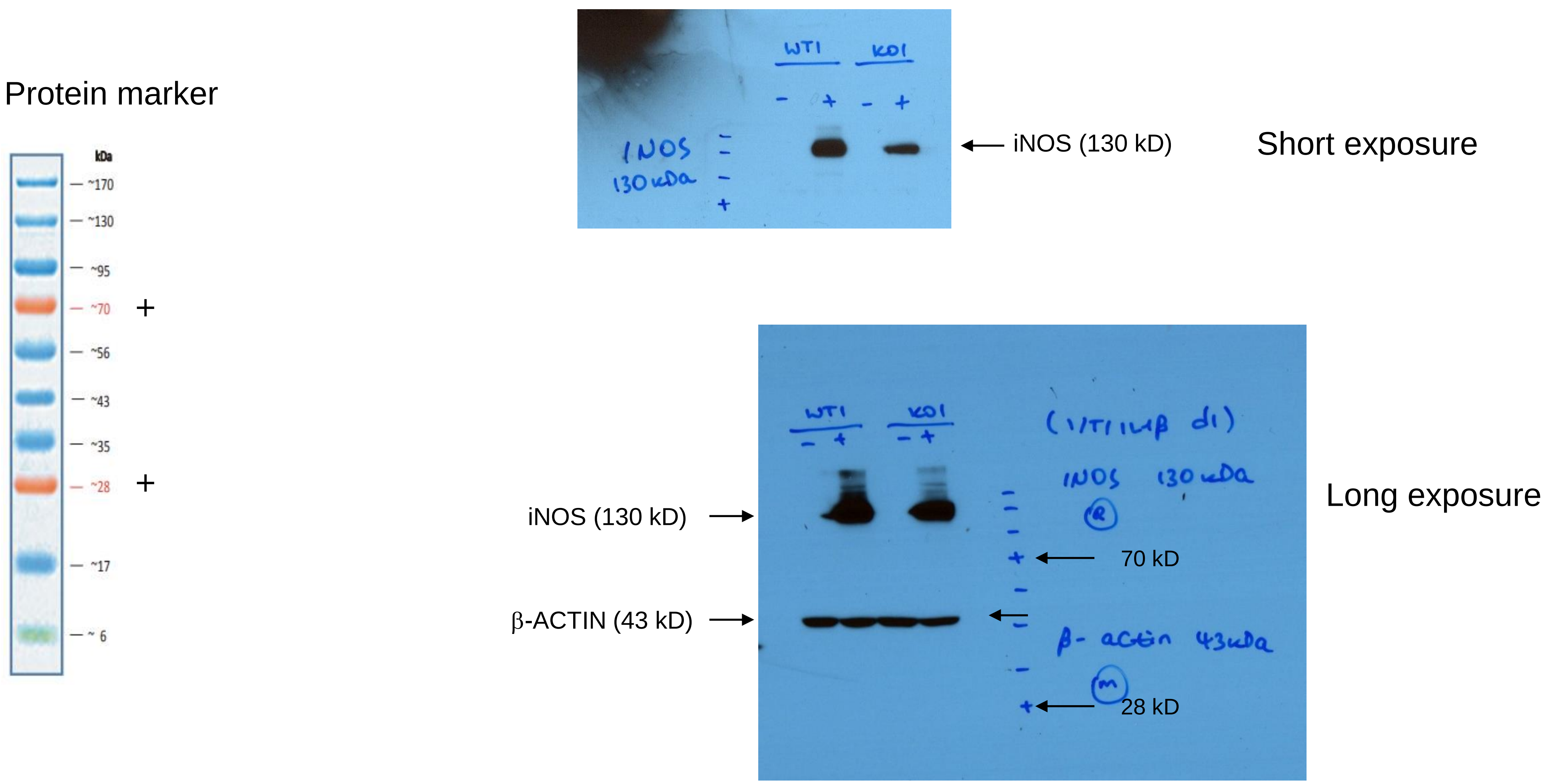

Protein marker
kDa
~170
~130
~95
~70
~56
~43
~35
~28
~17
~ 6
+
+
WT1
KO1
iNOS
130 kDa
iNOS (130 kD)
Short exposure
iNOS (130 kD)
β-ACTIN (43 kD)
70 kD
28 kD
β- actin 43kDa
Long exposure

Fig. 3E

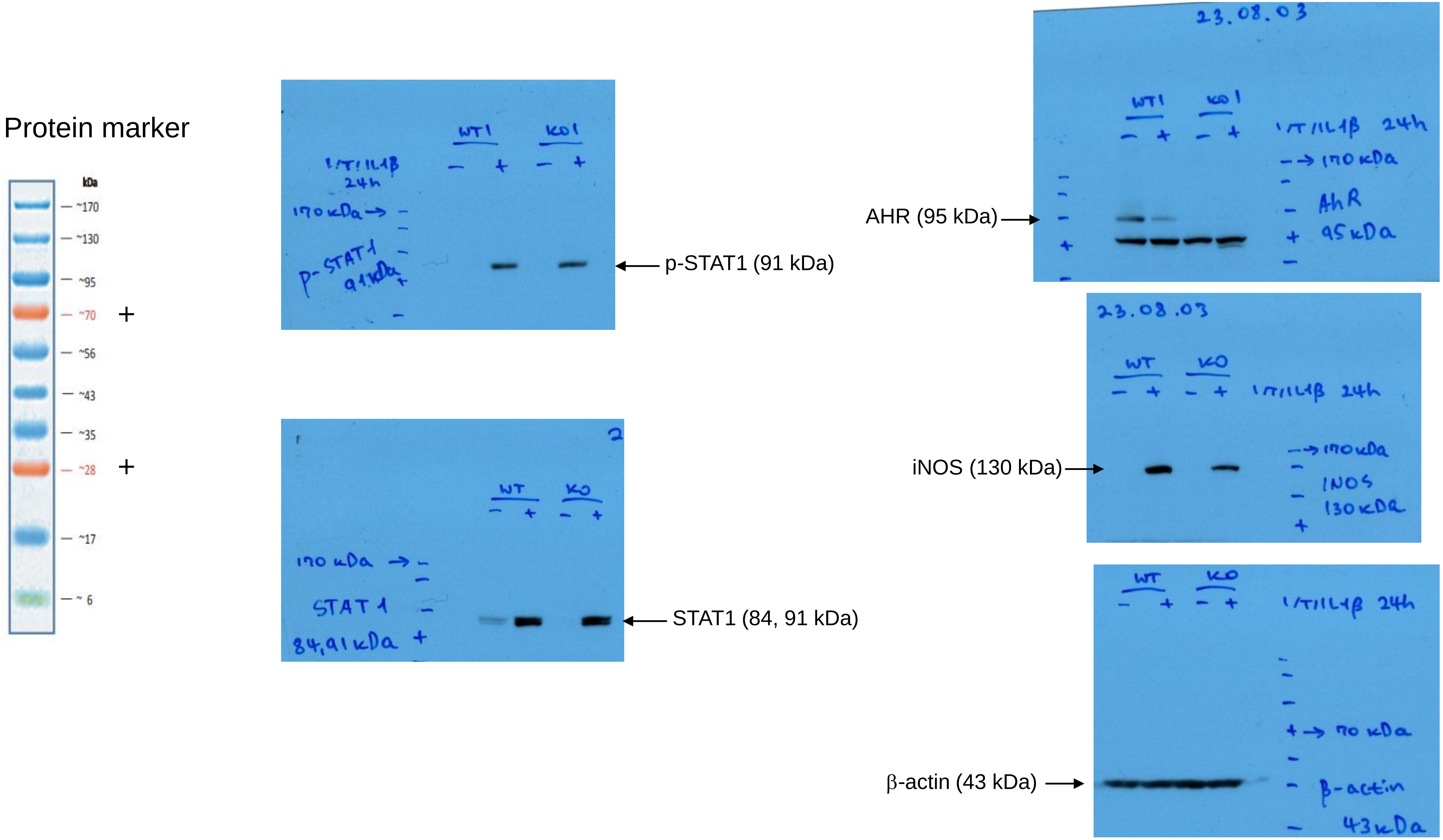
Protein marker
kDa
~170
~130
~95
~70
~56
~43
~35
~28
~17
~ 6
p-STAT1 (91 kDa)
STAT1 (84, 91 kDa)
AHR (95 kDa)
iNOS (130 kDa)
β-actin (43 kDa)

Fig. 4A

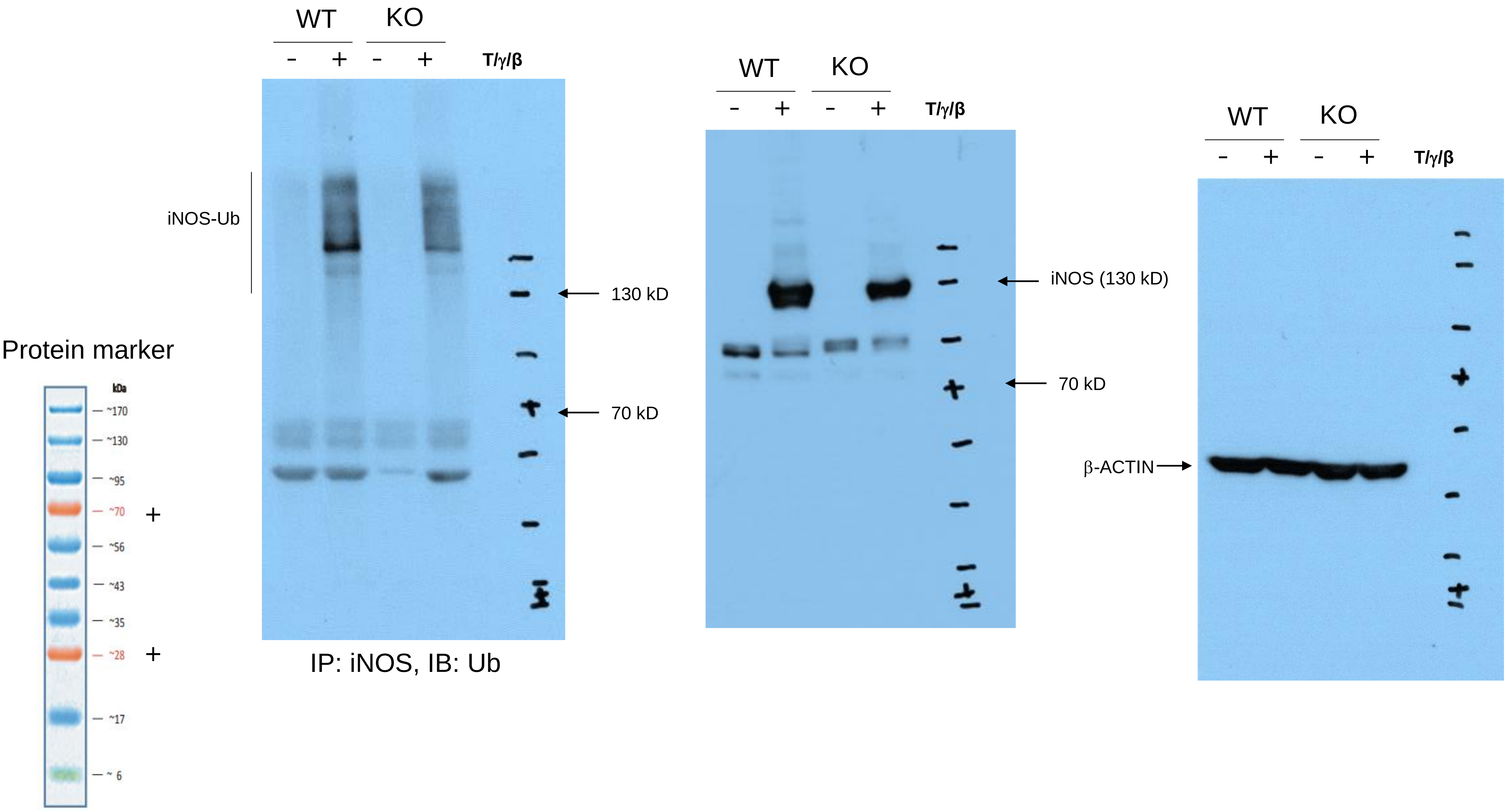
WT
KO
- + - + T/γ/β
iNOS-Ub
130 kD
70 kD
Protein marker
kDa
~170
~130
~95
~70
~56
~43
~35
~28
~17
~ 6
+
+
IP: iNOS, IB: Ub
WT
KO
- + - + T/γ/β
iNOS (130 kD)
70 kD
WT
KO
- + - + T/γ/β
β-ACTIN

Fig. 4B

Protein marker

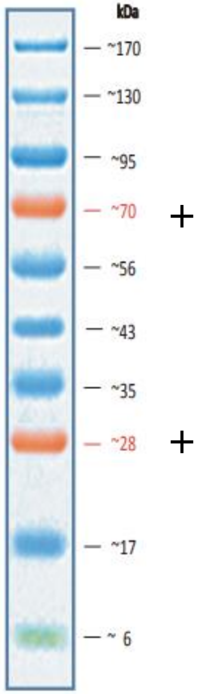

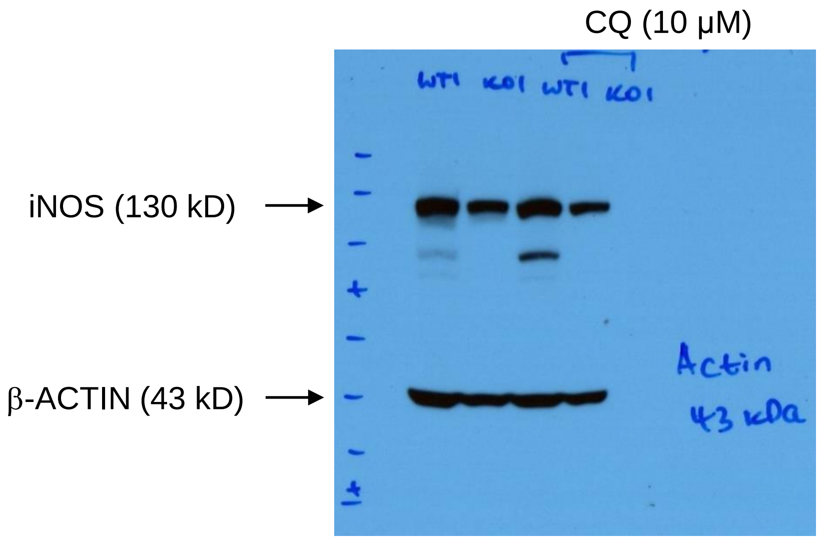

Fig. 4D

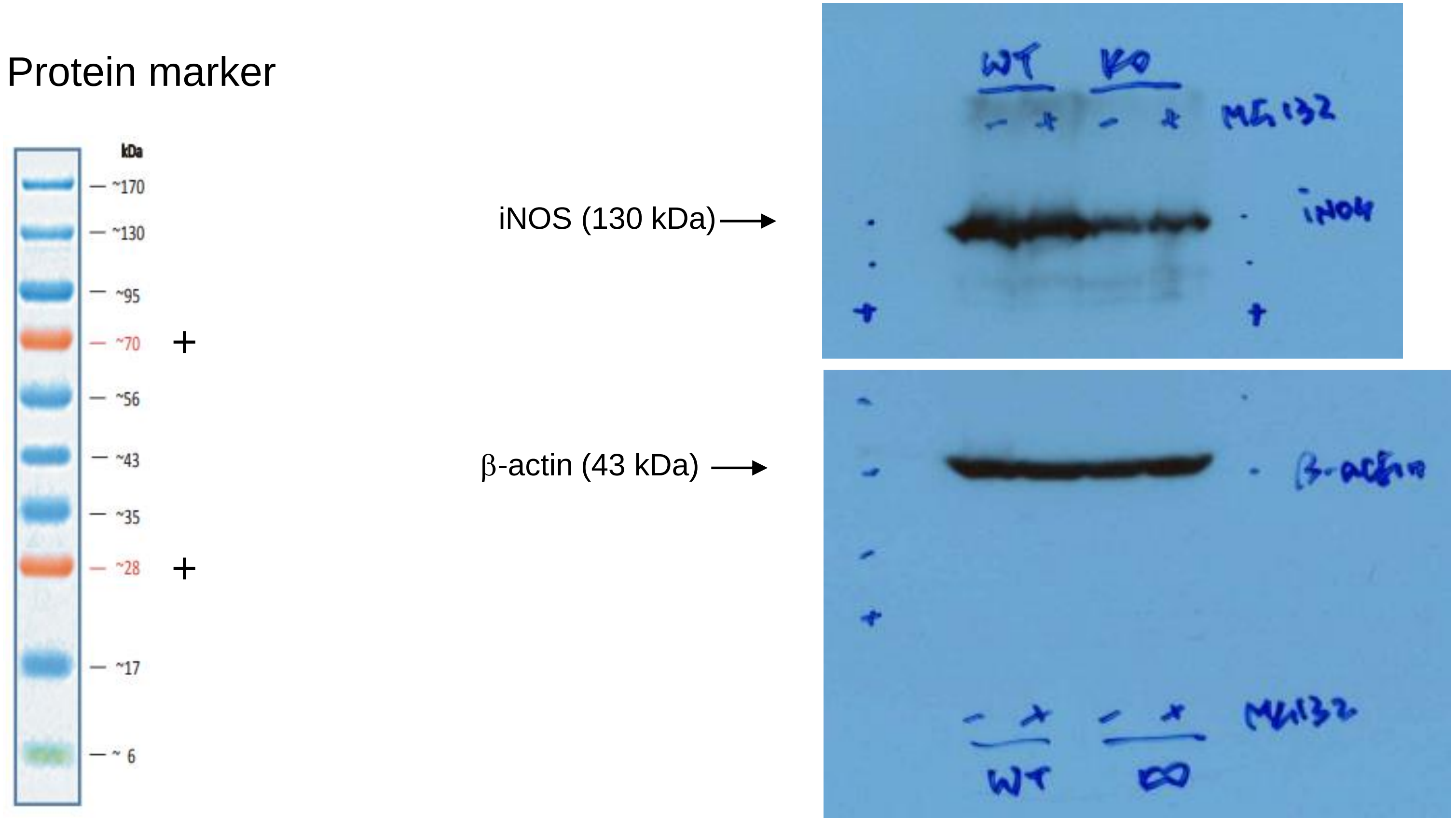

Protein marker
kDa
~170
~130
~95
~70
~56
~43
~35
~28
~17
~ 6
+
+
iNOS (130 kDa)
β-actin (43 kDa)
WT
KO
MG132
iNOS
β-actin

Fig. 5A

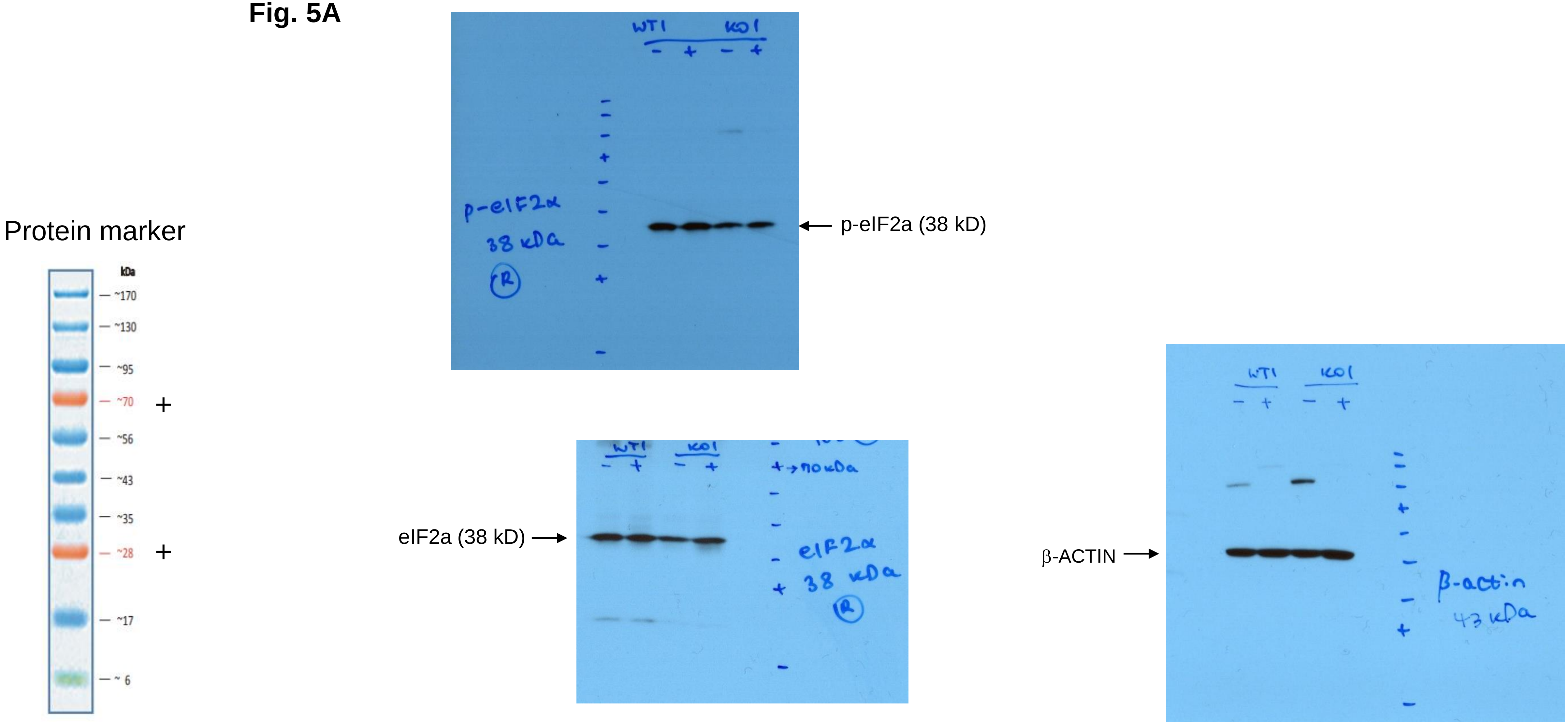

Protein marker
kDa
~170
~130
~95
~70
~56
~43
~35
~28
~17
~ 6
+
+
WT1
KO1
p-eIF2α
38 kDa
p-eIF2a (38 kD)
eIF2a (38 kD)
eIF2α
38 kDa
β-ACTIN
β-actin
43 kDa

Fig. 5B

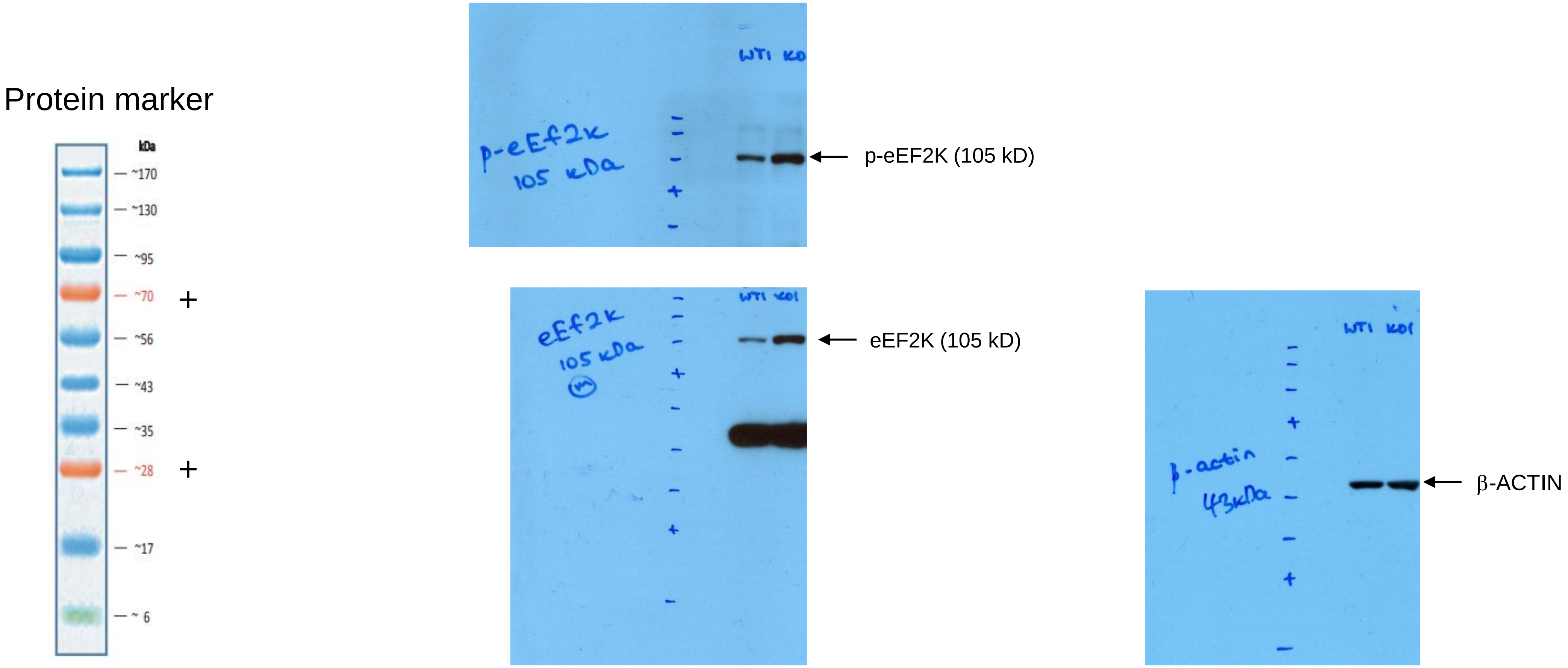

Protein marker
kDa
~170
~130
~95
~70
~56
~43
~35
~28
~17
~ 6
+
+
p-eEF2K (105 kD)
eEF2K (105 kD)
β-ACTIN

Fig. 5D

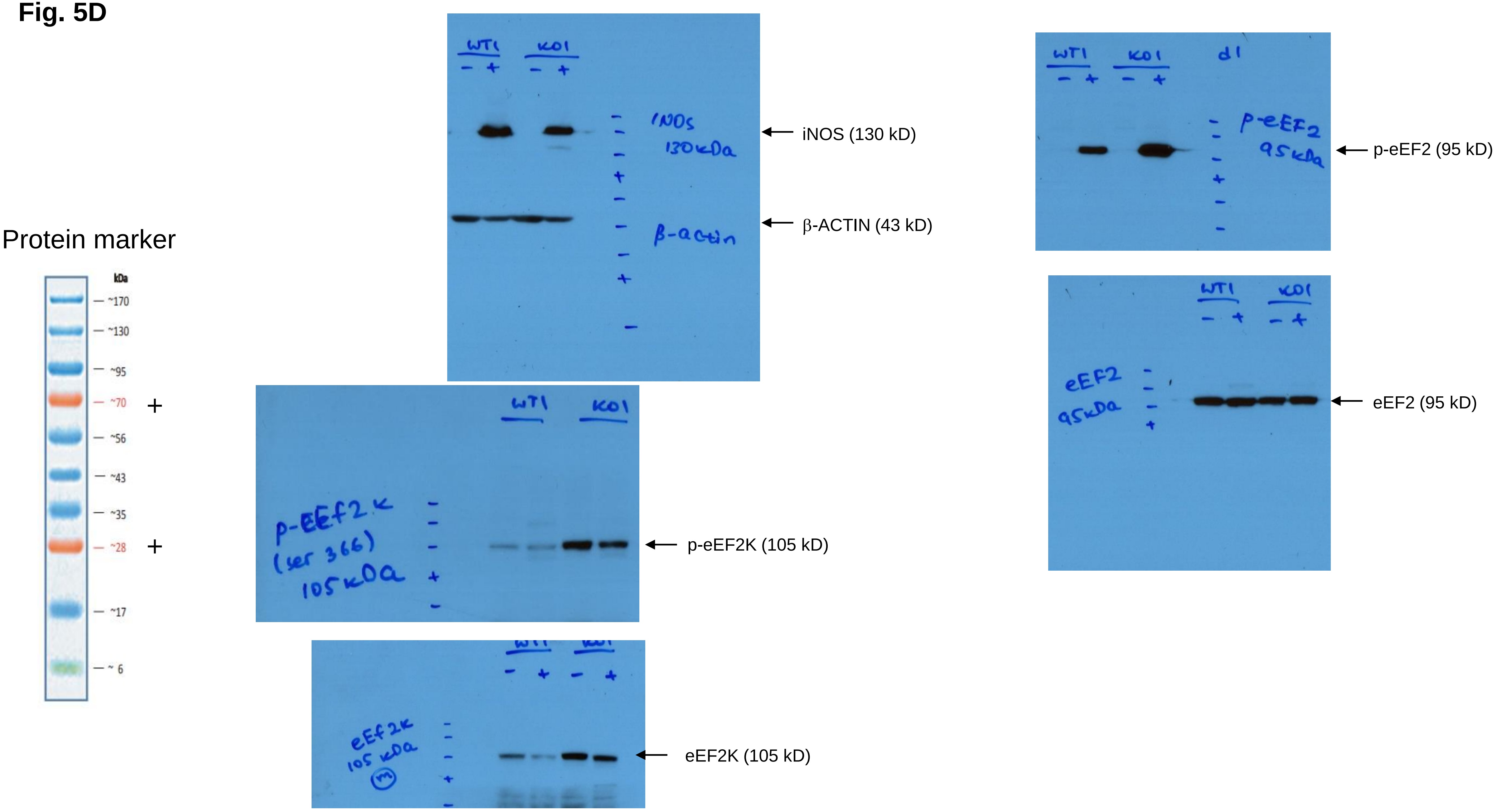

Protein marker
kDa
~170
~130
~95
~70
~56
~43
~35
~28
~17
~ 6
iNOS (130 kD)
β-ACTIN (43 kD)
p-eEF2 (95 kD)
eEF2 (95 kD)
p-eEF2K (105 kD)
eEF2K (105 kD)

Fig. 5E

Protein marker

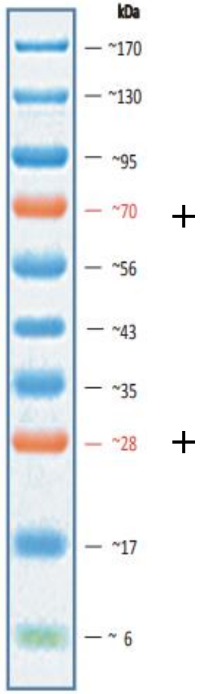

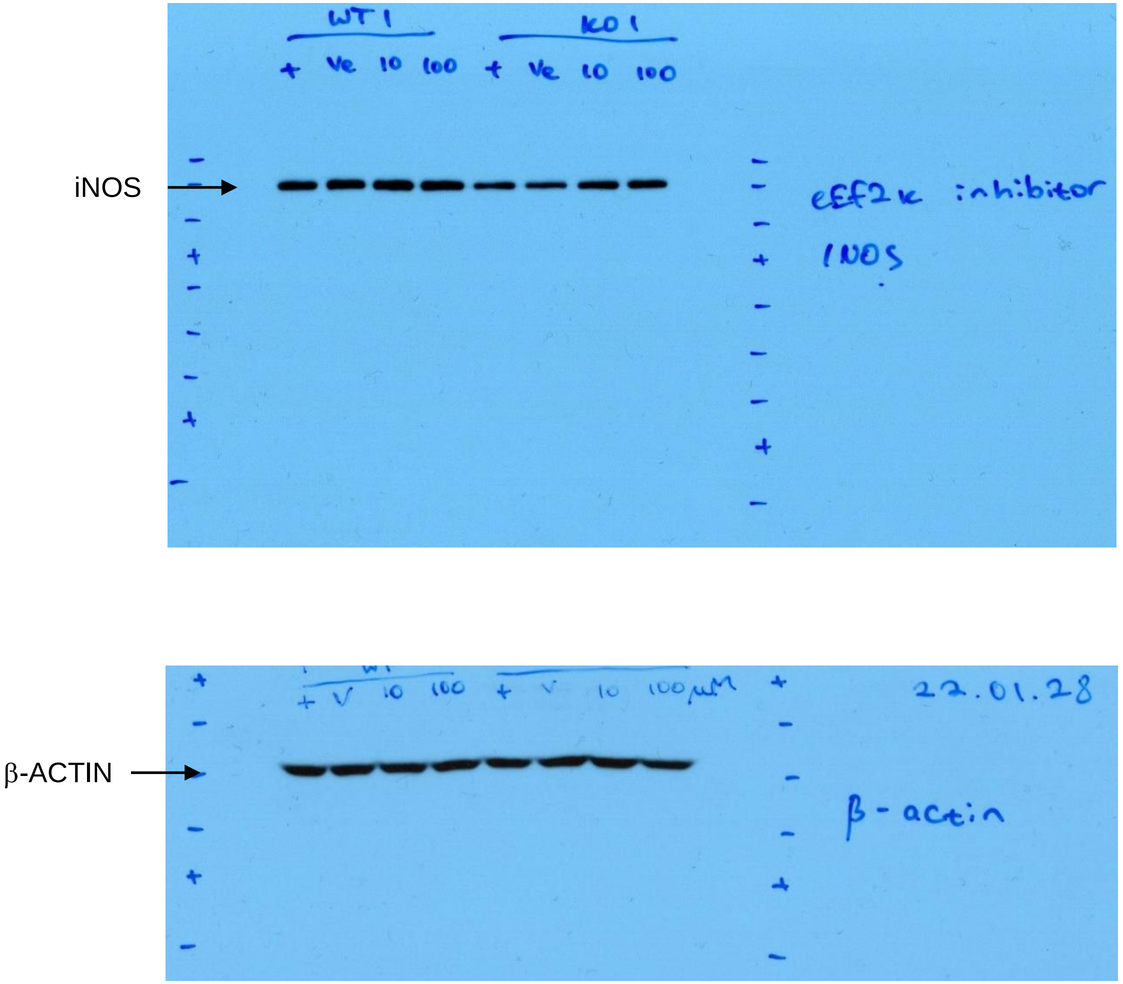

Fig. 6A

Protein marker

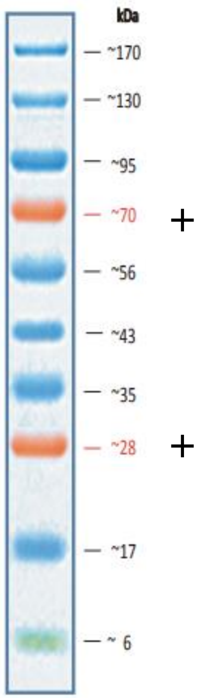

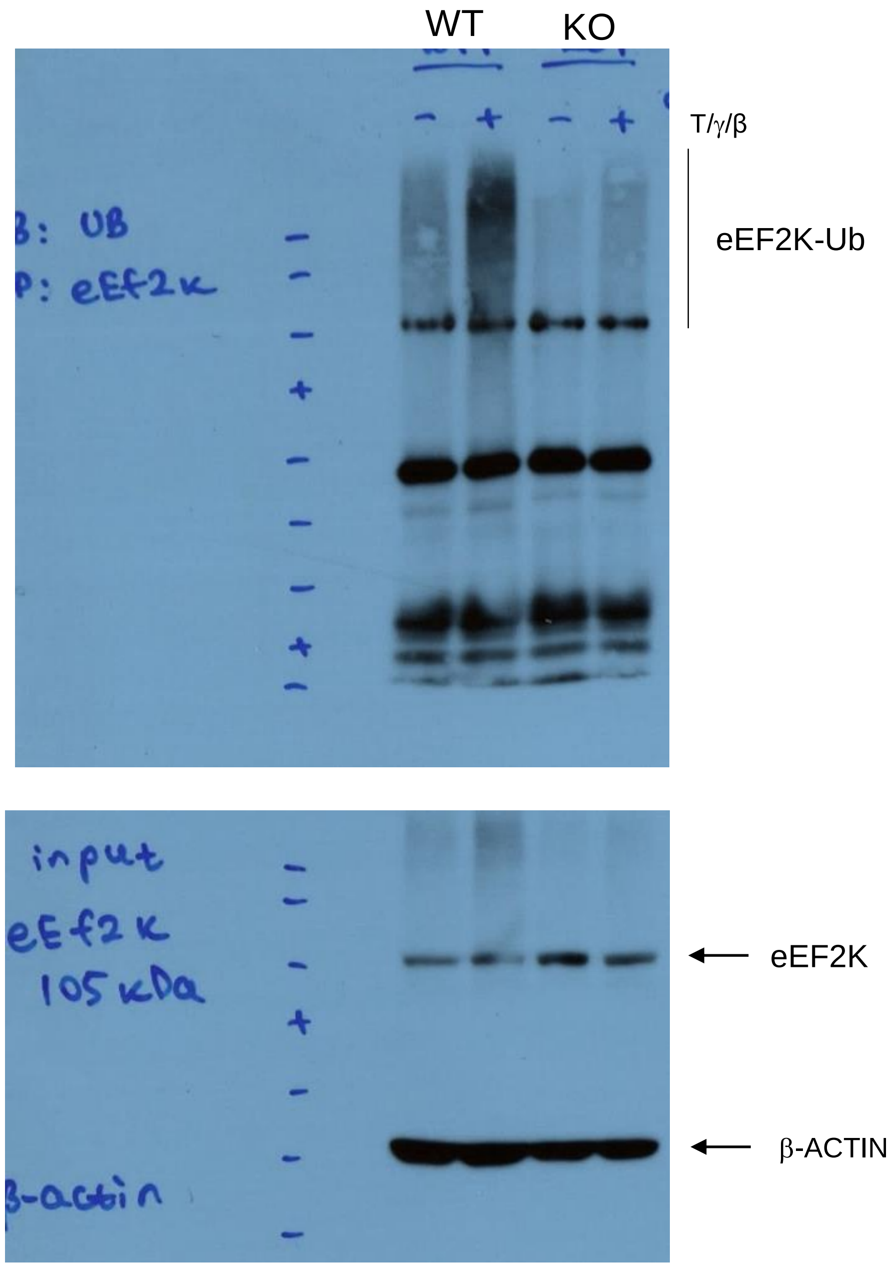

Fig. 6B

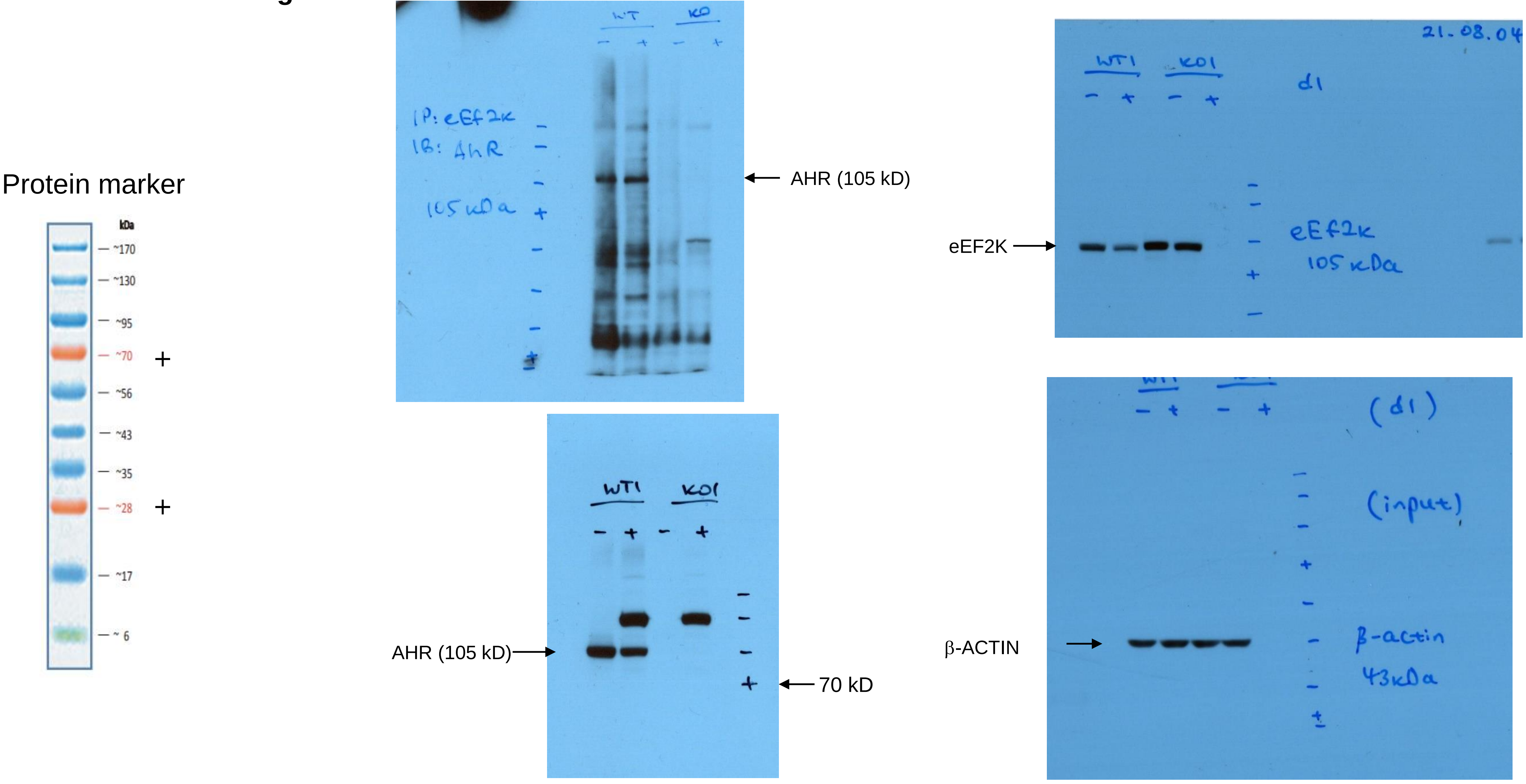

Protein marker
kDa
~170
~130
~95
~70
~56
~43
~35
~28
~17
~6
IP: eEF2K
IB: AhR
105 kDa
WT
KO
AHR (105 kD)
WT1
KO1
AHR (105 kD)
70 kD
21.08.04
d1
eEF2K
eEF2k
105 kDa
(d1)
(input)
β-ACTIN
β-actin
43kDa

Fig. 6C

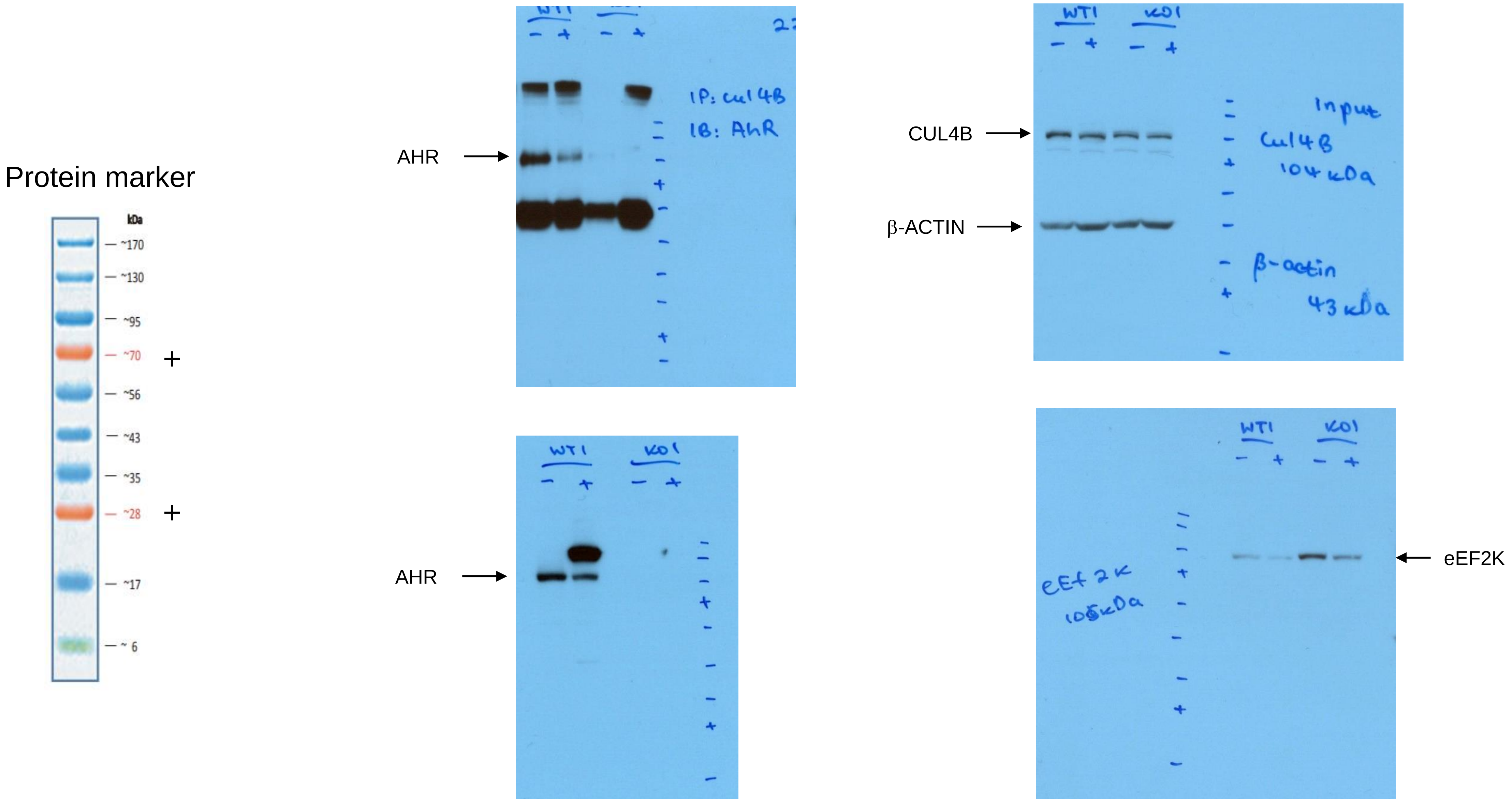
Protein marker
kDa
~170
~130
~95
~70
~56
~43
~35
~28
~17
~ 6
AHR
AHR
CUL4B
β-ACTIN
eEF2K
IP: Cul4B
IB: AhR
Input
Cul4B
104 kDa
β-actin
43 kDa

Fig. 6E

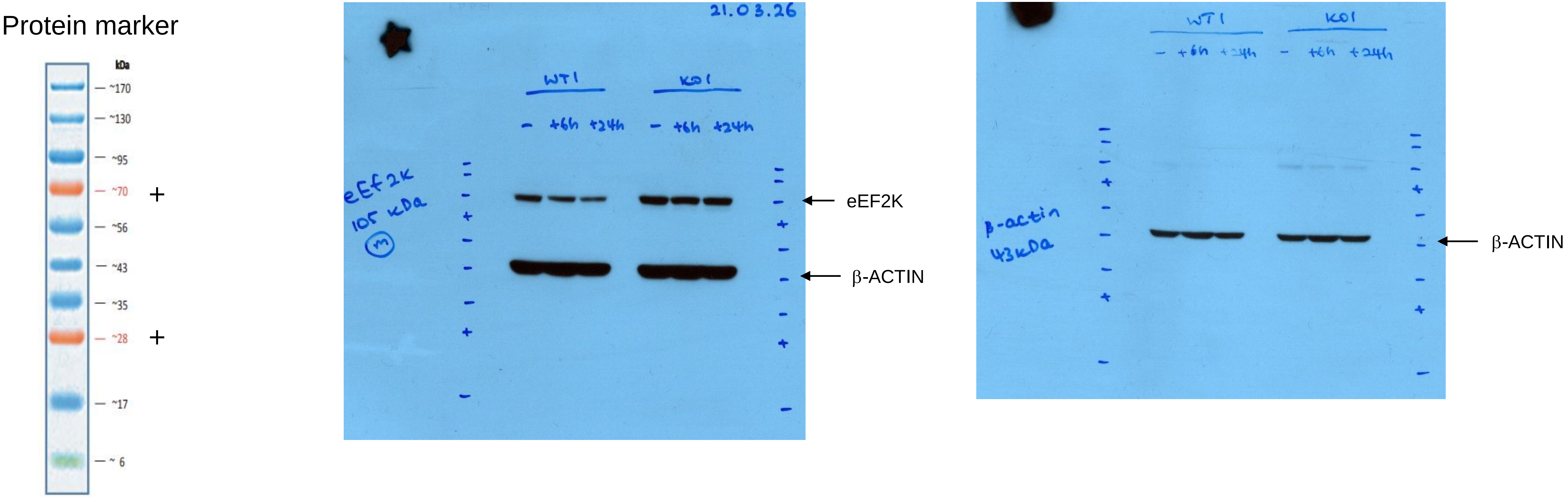
Protein marker
kDa
~170
~130
~95
~70
~56
~43
~35
~28
~17
~ 6
+
+
21.03.26
WT1
KO1
- +6h +24h
- +6h +24h
eEf2K
105 kDa
eEF2K
β-ACTIN
WT1
KO1
- +6h +24h
- +6h +24h
β-actin
43kDa
β-ACTIN

Protein marker

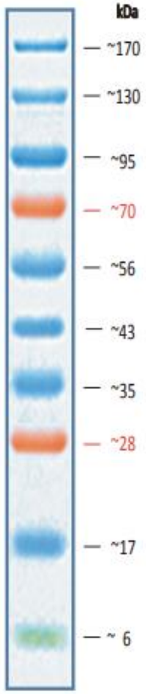

+

+

Fig. 7A

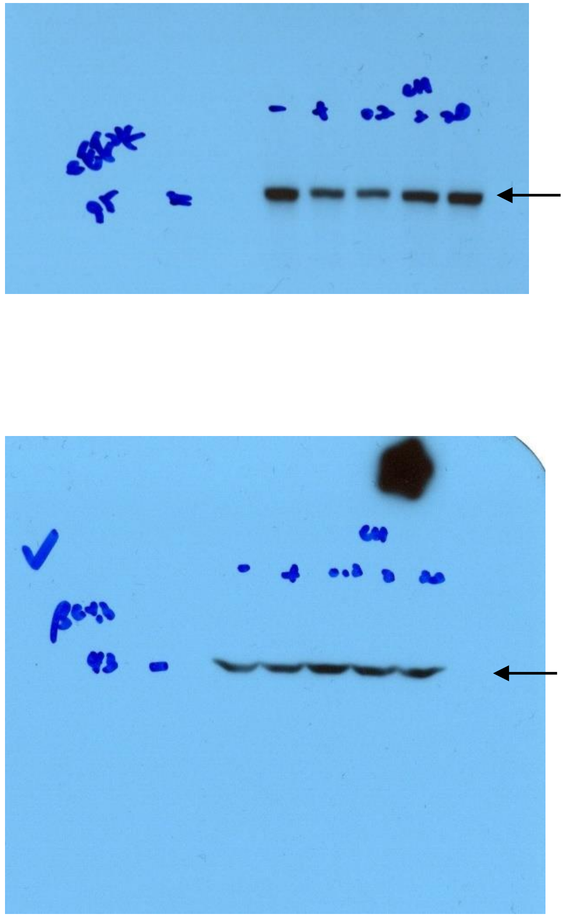

eEF2K

ACTIN

Fig. 7B

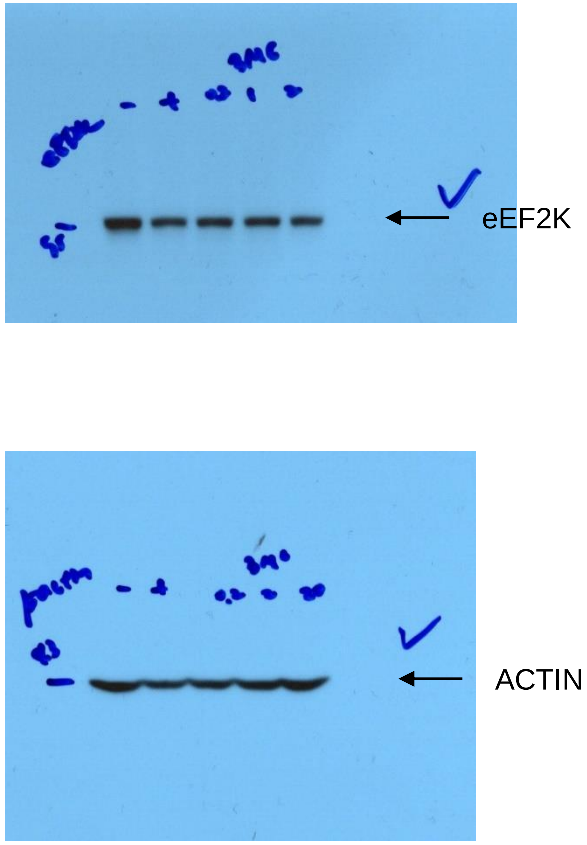

eEF2K

ACTIN

Fig. 7C

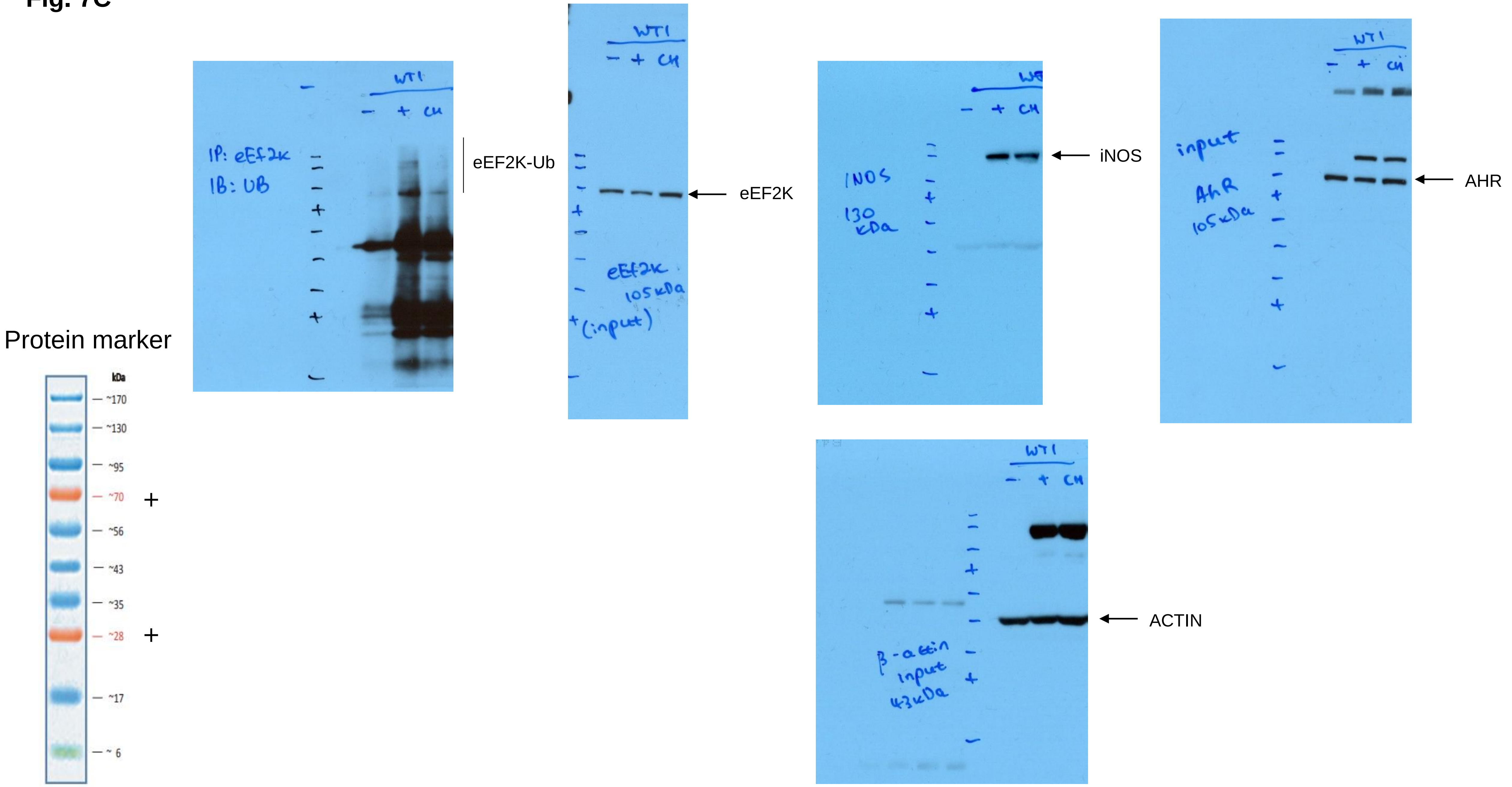
Protein marker
kDa
~170
~130
~95
~70
~56
~43
~35
~28
~17
~ 6
eEF2K-Ub
eEF2K
iNOS
AHR
ACTIN

Fig. 7D

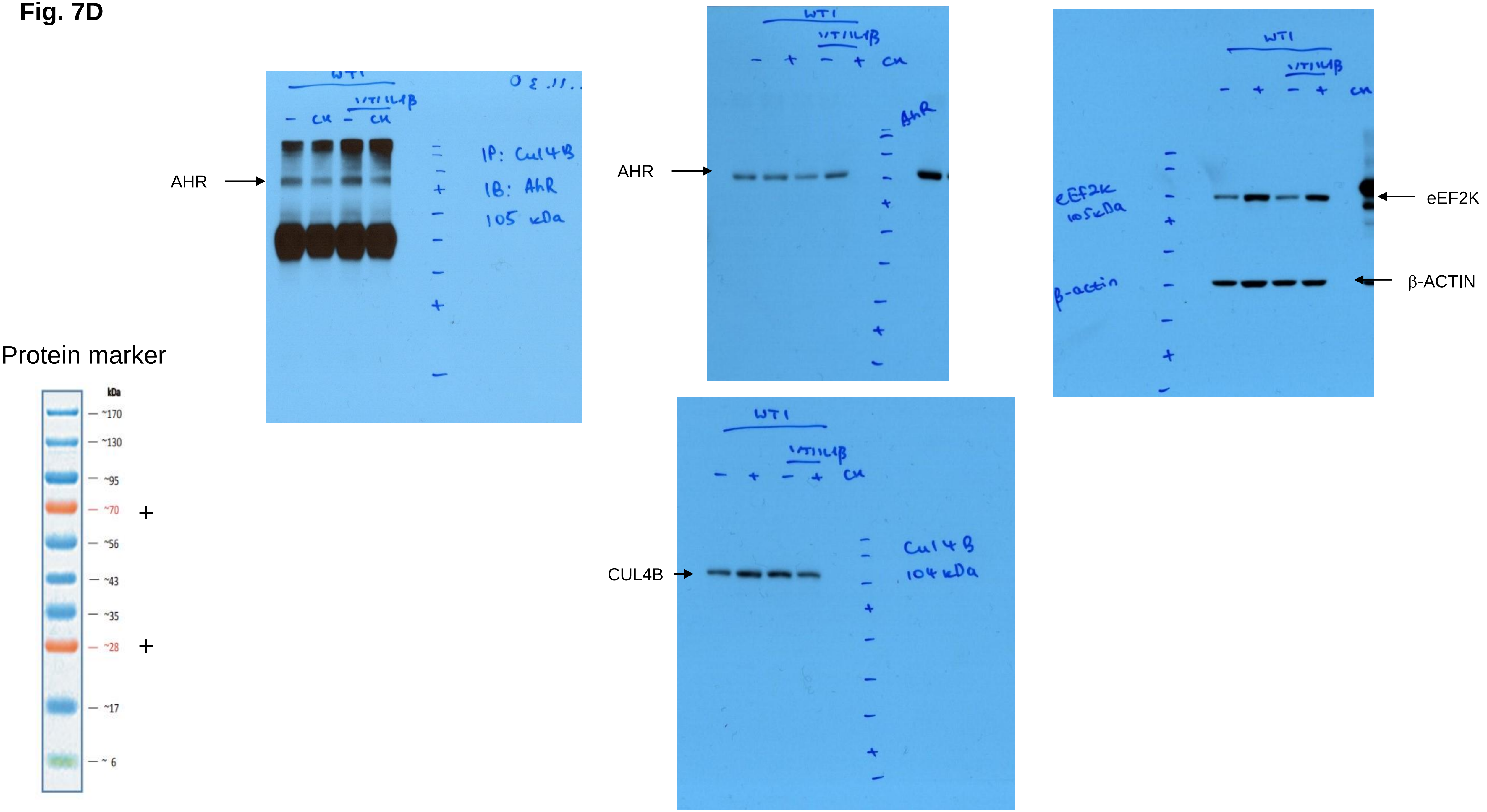
AHR
IP: Cul4B
IB: AhR
105 kDa
AHR
eEF2K
β-ACTIN
CUL4B
Protein marker
kDa
~170
~130
~95
~70
~56
~43
~35
~28
~17
~ 6

Fig. 7E

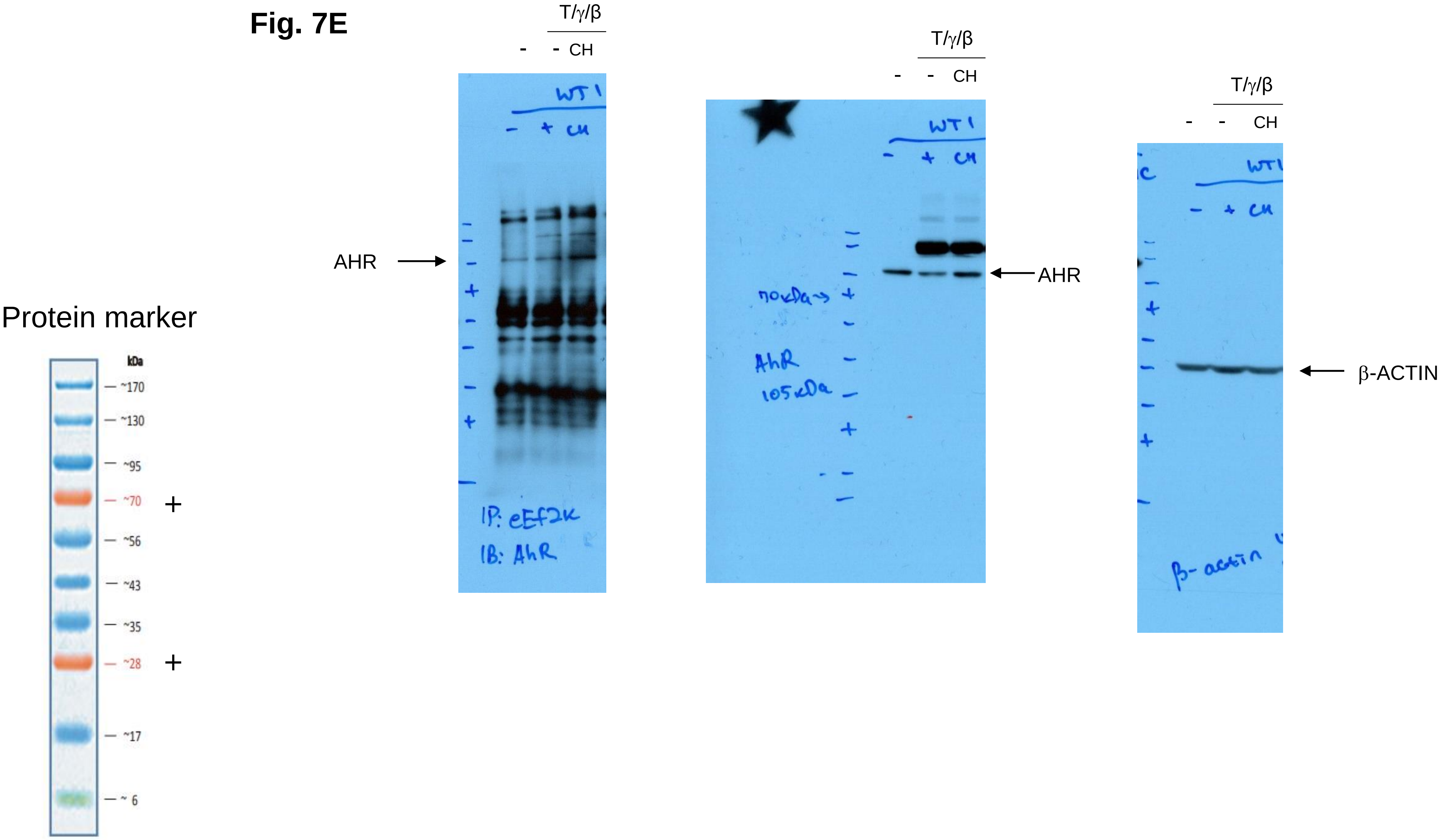
T/γ/β
- - CH
WT1
- + CH
AHR
IP: eEf2K
IB: AhR
70kDa
AhR
105kDa
β-ACTIN
β-actin
Protein marker
kDa
~170
~130
~95
~70
~56
~43
~35
~28
~17
~6

Fig. 8A

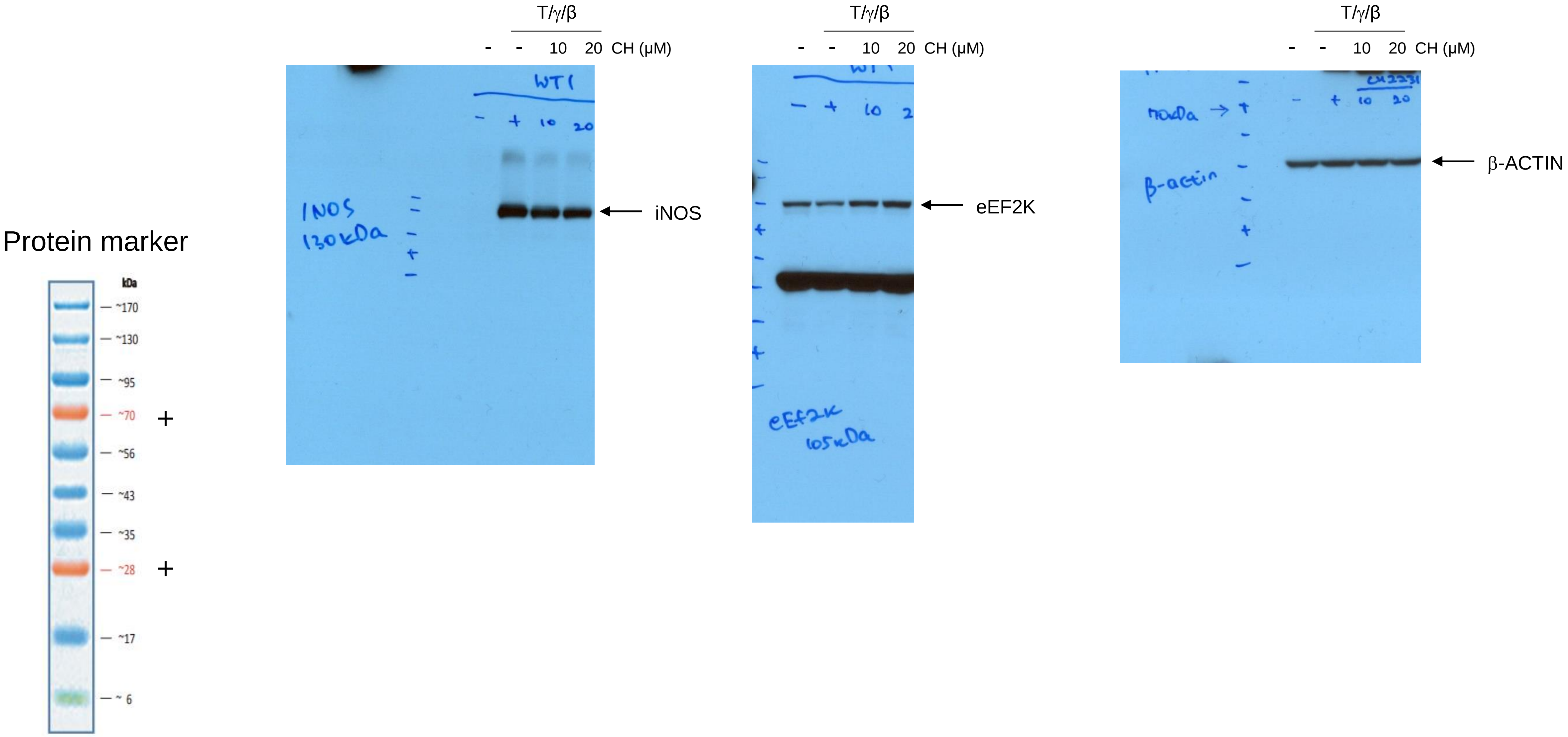
T/γ/β
- - 10 20 CH (μM)
T/γ/β
- - 10 20 CH (μM)
T/γ/β
- - 10 20 CH (μM)
iNOS
eEF2K
β-ACTIN
Protein marker
kDa
~170
~130
~95
~70
~56
~43
~35
~28
~17
~ 6

Supplementary Fig. 2

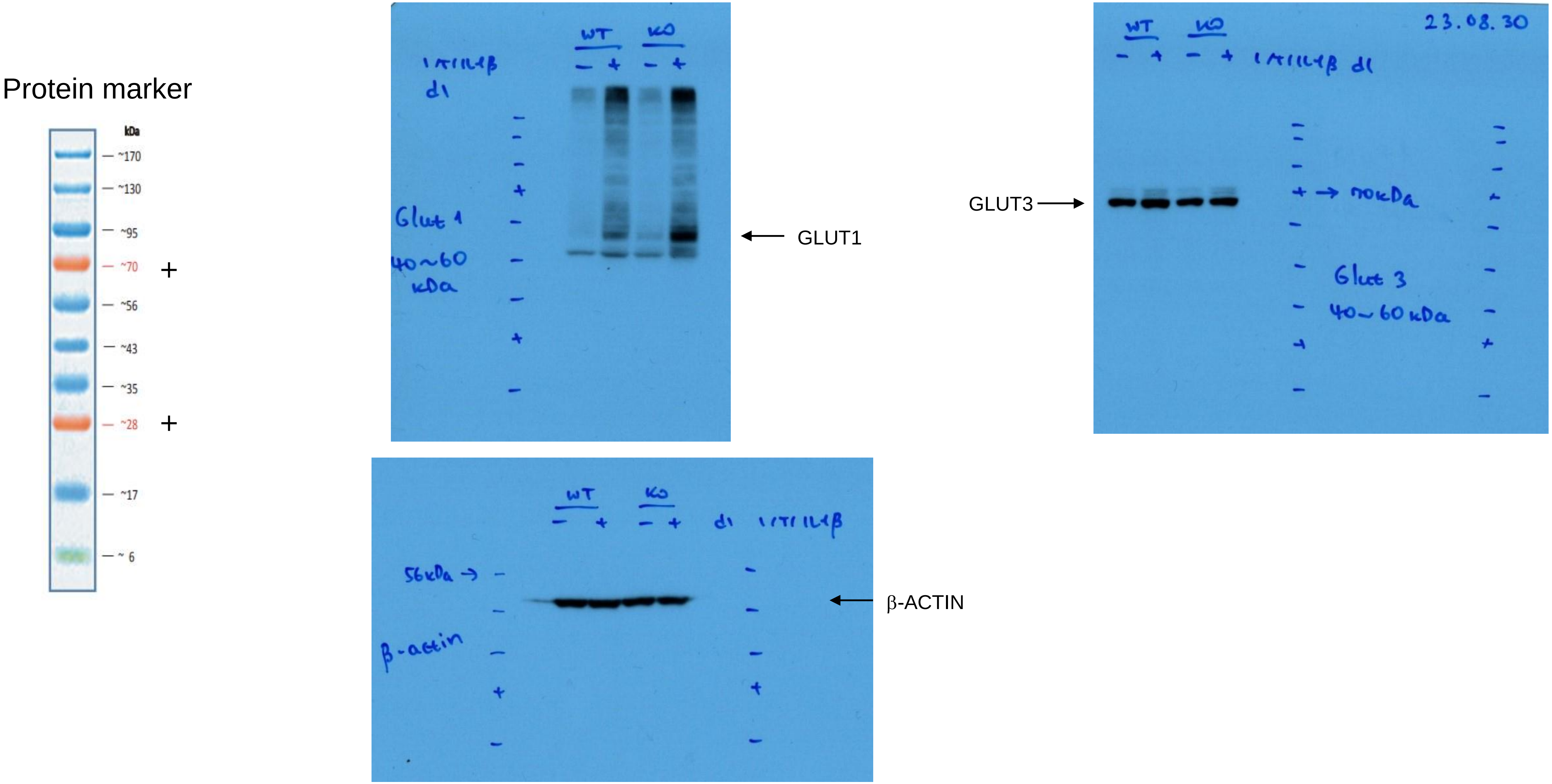

Supplement: Supplementary file 2 — Original Data File [file 41419_2023_6341_MOESM2_ESM.pdf]
